# Global patterns and inequalities in healthy aging: a multidimensional analysis using composite index and explainable AI

**DOI:** 10.3389/fpubh.2026.1841479

**Published:** 2026-06-05

**Authors:** Sadullah Çelik, Cemile Zehra Köroğlu

**Affiliations:** 1 Department of International Trade and Finance, Nazilli Faculty of Economics and Administrative Sciences, Aydin Adnan Menderes University, Aydin, Türkiye; 2 Department of Social Work, Faculty of Economics and Administrative Sciences, Uşak University, Uşak, Türkiye

**Keywords:** clustering, composite index, explainable artificial intelligence, healthy aging, inequality, principal component analysis, public health, shap

## Abstract

**Background:**

Population aging is a global phenomenon with significant implications for public health systems. Understanding the multidimensional determinants of healthy aging is essential for developing effective and equitable policies across countries.

**Methods:**

This study develops a composite Healthy Aging and Prevention Index (HAPI) to assess global healthy aging patterns using indicators of life span, health span, work span, income, environmental performance, and happiness. Missing data are addressed using Multiple Imputation by Chained Equations (MICE). Principal Component Analysis (PCA) is employed to construct the composite index, while entropy weighting determines the relative contribution of each dimension. K-Means clustering is applied to group countries with similar healthy aging profiles. To improve interpretability, SHapley Additive exPlanations (SHAP) are used to quantify the contribution of each variable. Inequality in healthy aging outcomes is assessed using the Gini coefficient and Lorenz curve.

**Results:**

The findings indicate that life span, health span, income, environmental performance, and happiness are the primary drivers of healthy aging, while work span has a relatively limited contribution. Cluster analysis reveals substantial heterogeneity in healthy aging patterns across countries. Inequality analysis shows moderate but notable disparities in HAPI scores (Gini = 0.318), suggesting uneven distribution of healthy aging outcomes globally.

**Conclusion:**

Healthy aging is a multidimensional construct shaped largely by socio-economic and environmental determinants rather than healthcare factors alone. These findings highlight the need for context-specific and integrated public health strategies tailored to country clusters. The proposed framework provides a robust and interpretable tool for policymakers to evaluate and improve healthy aging outcomes at the global level.

## 1 Introduction

The fast-aging world population has made healthy aging and preventive health care more prominent in public health studies and policies. While increasing life expectancy is not sufficient in modern society, it is now equally important to maintain a healthy state of well-being in later life. As a result, there is a growing need to close the gap between life expectancy and healthy life expectancy, which is a major challenge in global health studies.

To assess healthy aging, a multidimensional method is necessary to incorporate demographic, socio-economic, environmental, and well-being variables. While conventional health studies use health outcomes like mortality and morbidity to assess health, recent studies have suggested that overall well-being is a critical aspect of health. In this regard, composite measures are a powerful tool to assess healthy aging in a population. For example, regional healthy aging scales have been proposed to assess functional ability and intrinsic capacity in a population; however, most of these methods are limited to a specific geographical location and lack global analysis (1).

This research is based on the use of the Healthy Ageing Prevention Index, which is a multidimensional approach to measuring healthy aging performance through the use of indicators such as life expectancy, healthy life expectancy, working life duration, income level, environmental performance, and happiness. Previous research has shown that such multidimensional indices are strongly correlated with long-term outcomes such as quality of life, dependency, and mortality risks (2).

What makes the current research novel is the incorporation of artificial intelligence and data science approaches into the HAPI framework. In particular, the research relies on the use of PCA for dimension reduction and the calculation of a composite healthy aging score, and on the use of K-Means clustering for grouping countries with similar health and well-being profiles. Moreover, the research also relies on the use of SHAP, which is used to provide an interpretable perspective on the use of machine learning for the calculation of the global healthy aging score.

Through the use of dimensionality reduction techniques, unsupervised learning, and explainable AI, this study aims to propose a data-driven analytical framework in examining global healthy aging patterns. The study's findings contribute to healthy aging and data science fields by establishing structural differences between countries and explaining the multi-dimensional factors that influence healthy aging outcomes.

In addition to dimension reduction and clustering techniques, the study will also adopt the entropy weighting method for the HAPI index. The entropy weighting method will enable the measurement of the level of importance of each feature, such as life expectancy, healthy lifespan, income, environmental performance, duration of working life, and happiness, in determining the healthy aging score. This is a significant improvement in the robustness of the composite index, as it considers the variability and information content of each feature across nations. The PCA, K-Means clustering, and entropy weighting methods will collectively provide a comprehensive framework for measuring healthy aging, considering the structural differences and multifaceted nature of the concept of well-being from a global perspective.

The remainder of the paper is structured as follows: Section 2 discusses the theoretical foundations of healthy aging. Section 3 discusses the data and methodologies used in this study. Section 4 presents the empirical findings of this study. Finally, in Section 5, policy implications and future research directions are discussed.

## 2 Theoretical framework and literature review

The idea of healthy aging is thought to be multidimensional and not only dependent on the length of one's life and is associated with physical, functional, and social well-being (3). The definition of healthy aging according to the theory of functional ability developed by the World Health Organization is connected to the possibility of older adults retaining functional ability, mobility, and independence in old age (4). It represents the transition from the traditional medical approach to deficits to a new understanding based on the interplay between a person's intrinsic capacity and their surrounding environment (4, 5). Healthy aging is viewed as an outcome of biological, socio-economic, and environmental exposures during people's life course, and intrinsic capacity is identified as a strong predictor of future care needs and death (6).

Health promotion interventions are regarded as essential in contributing to healthy aging and reducing the risks of developing chronic diseases (7). Empirical research has shown that healthy habits are positively correlated with sustaining functional capacity during aging (8). In addition to personal lifestyle choices, the concept of a “social gradient” reveals that people who live in more economically stable environments show signs of functional impairment at a slower rate (9). Moreover, environmental factors have been determined to play an important role in healthy aging, affecting inflammatory responses and psychological well-being (10).

The results from comparative international research have found remarkable variations in healthy aging across different countries. The application of multidimensional scales, including the ATHLOS Healthy Aging Scale and regional healthy aging index, has revealed considerable correlations between healthy aging measures and mortality rates (2, 11). However, although these scales play an important role in offering information about healthy aging among nations, their limitations lie in their inability to adequately reflect non-linear interaction in varied socioeconomic environments (1).

Over the past few years, data science and machine learning (ML) have provided new possibilities for studying multidimensional health-related data (12). Techniques in statistical learning can help uncover relationships between structural variables by applying techniques such as PCA to multidimensional health data, while clustering can classify nations based on similar structural characteristics using approaches like K-Means clustering (13). The problem of ML being a “black box” can be resolved by applying XAI techniques such as SHAP, which enables consistent attribution of features (14). Such transparency is important to ensure that ML-based prediction models can be relied upon when considering global health governance issues (15).

The use of multi-dimensional measures of health, together with machine learning and explainable artificial intelligence techniques, allows this research to provide a comprehensive methodology for assessing the dynamics of healthy aging worldwide while recognizing the structural determinants of wellbeing among countries. The adoption of the entropy-weighting algorithm and the utilization of SHAP-based decompositions help fill some of the deficiencies present in the existing literature, which considers the phenomenon of aging as a medical issue without addressing its social and structural nature (16). Not only does this methodology enable the identification of inequality dynamics, but it also serves as a powerful instrument for policy simulations.

## 3 Data and methods

**Figure 1:**
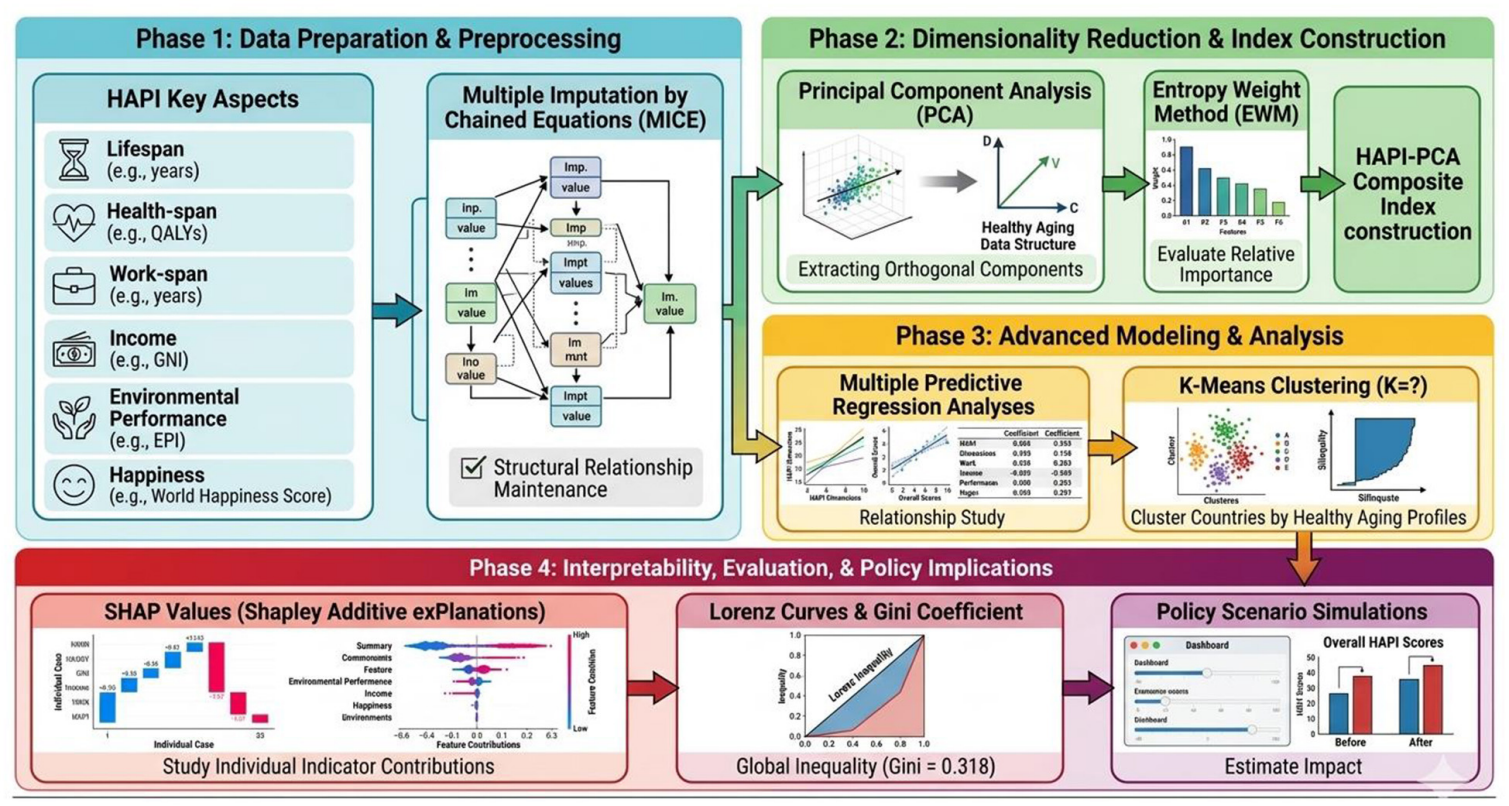
Methodological framework and data workflow.

The methodological framework of the study, as presented in Figure 1 below, uses a structured multi-stage method to evaluate the concept of global healthy aging through the application of the HAPI. The first phase of the study is dedicated to the preparation and preprocessing of the data by incorporating key aspects such as lifespan, health-span, work-span, income, environmental performance, and happiness. The missing values are handled through the MICE method. This method ensures the maintenance of the structural relationship between the data. The second phase of the study incorporates the dimensionality reduction and index construction. In this case, the PCA method is applied to extract orthogonal components based on the data structure of healthy aging. The entropy weight method is applied to evaluate the relative importance of the features, ensuring a composite index of HAPI-PCA on the outcomes of healthy aging.

Phase 3 and Phase 4 further expand the study by considering advanced modeling techniques and interpretability. In Phase 3, multiple predictive regression analyses are performed to study the relationship between HAPI dimensions and overall scores. K-Means clustering is performed on the countries to cluster them according to healthy aging profile features. Phase 4 mainly deals with the interpretability of the results. SHAP values are calculated to study the contribution of individual indicators. Lorenz curves are plotted to study global inequality (Gini coefficient = 0.318). Policy scenario simulations are performed to estimate the impact on overall HAPI scores. The entire workflow is transparent and robust. It provides a solid foundation for healthy aging research.

### 3.1 Data

The current study uses the HAPI dataset that gives comparative data from a global sample of 153 countries. The HAPI dataset includes the indicators of six dimensions: life expectancy (life span), healthy life expectancy (health span), work time (work span), income, environmental performance, and happiness (17). Particularly, health span is measured as the mean years an individual is healthy without any illnesses or disabilities, thereby contrasting it with life span. In the case of the HAPI dataset, despite its wide range of countries covered, zero values were taken as missing data and not as actual measurements. This was to ensure that the sample size of 153 countries remained intact. To prevent any biases, missing data in the dataset were imputed through the use of MICE.

The main factors used in determining the HAPI index were obtained from the databases on the ILCUK Healthy Aging and Prevention Index and the WHO Global Health Estimates. The life expectancy factor means the average years that can be lived assuming mortality rates will stay the same throughout the lifespan of a baby. The healthy life expectancy (HALE) was computed using the Sullivan Method, which adjusts the average life expectancy based on the years lived in sickness or disability.

The cross-country healthy aging data and Python code used in the analysis of this paper, and all of the HAPI scoring results, are shared. The codes related to the use of sophisticated analysis techniques such as PCA analysis and regression analysis, are available at GitHub. In light of open science and openness, the whole process of the analysis performed in this paper is accessible and available on GitHub at https://github.com/Sadullah4535/Healthy-Aging-and-Preventive-/upload/main.

### 3.2 Data preprocessing and missing data filling

The data set contains various indicators related to healthy aging and socio-economic factors in different countries. As is the case with many data sets containing information from different countries, there is a possibility that some data could be missing due to the way the data is reported in the respective countries. The missing values were filled using the MICE method.

If the data matrix containing information on *n* countries and *p* indicators is represented as *X*, it can be represented as follows:


$$\begin{array}{l} X=\left[\begin{array}{ccc} \begin{array}{c} x_{11}\\ x_{21} \end{array} & \begin{array}{c} x_{12}\\ x_{22} \end{array} & \begin{array}{c} \begin{array}{cc} \ldots & x_{1p} \end{array}\\ \begin{array}{cc} \ldots & x_{2p} \end{array} \end{array}\\ \vdots & \vdots & \begin{array}{cc} \ddots & \vdots \end{array}\\ x_{n1} & x_{n2} & \begin{array}{cc} \ldots & x_{np} \end{array} \end{array}\right] \end{array}$$

In this method, each missing value is imputed iteratively based on the results of regression models conditioned on other observed variables. MICE is an algorithm that creates several imputed datasets and combines the estimates to address the uncertainty in the imputation (18).

In the summary of the results, it is found that the distribution of the problem of missing data is not uniform for all the indicators. There were no missing data in the variables of life expectancy, healthy life expectancy, and working life, but in the income variable, the percentage of missing data is found to be 1.96%; in the environmental performance variable, the percentage is 3.92%; and in the variable related to the happiness ness, the percentage is 6.54%.

### 3.3 Principal component analysis

Principal component analysis (PCA) was used to simplify the complex set of various relationships between the different HAPI dimensions. PCA is a multivariate analysis technique that translates the shared variance in multivariate data sets into new orthogonal components called principal components (19). PCA makes it possible to discover the sources of the basic variance in high-dimensional data sets by reducing the data complexity and making the differences and similarities in the data sets easy to analyze. This is an imperative step in the analysis, especially in cross-country analysis, for the purposes of visualizing the impact of various combinations of the different components in the total score for healthy aging (20).

Mathematically, PCA is done on the standardized data matrix **X**∈*R*^*n*×*p*^ as given below:

- *Data standardization*: as given in Equation 1, each variable is normalized so that its mean is 0 and its standard deviation is 1.

$$\begin{array}{l} {\text{Z}}_{\text{i}\text{j}}=\frac{X_{\text{i}\text{j}}-\mu_{j}}{\sigma_{j}} \end{array}$$ (1)

In Equation 1, μ_*j*_ represents the mean value of each column and σ_*j*_ represents its standard deviation.

- *Calculating the covariance matrix*: in PCA analysis, the covariance matrix **Σ** is calculated from the standardized data matrix as in Equation 2.

$$\begin{array}{l} \Sigma =\frac{1}{n-1}{\text{Z}}^{T}\text{Z} \end{array}$$ (2)

- *Obtaining Eigenvalues and Eigenvectors*: the eigenvalues of the covariance matrix (λ_1_, λ_2_, …, λ_*p*_). These eigenvalues correspond to the eigenvectors (*v*_1_, *v*_2_, …, *v*_*p*_). Here, the eigenvectors determine the direction of the new components.
- *Creating new components*: in PCA analysis, the first *k* components explain the majority of the total variance and are calculated as in Equation 3.

$$\begin{array}{l} \text{P}{\text{C}}_{k}=\text{Z}{\text{v}}_{k}\;\;for\;\;i=1,2,\ldots ,k \end{array}$$ (3)

In this manner, the *PC*_1_, *PC*_2_, …, *PC*_*k*_ HAPI indicators are extracted as principal components that capture, in a summarized form, the multidimensional structure. The strength of PCA is that it is capable of transforming the dimensionality of the information while at the same time retaining its structure of correlation. This is of paramount significance, especially regarding input in policy analysis and models of clustering. PCA is proven in the literature (21).

Although the “work span” indicator has a statistically low correlation with health and wealth, it is considered an important pillar of the HAPI index. In theory, work span plays an essential role as a proxy measure for social engagement as well as functional ability of the older adults, as per the multidimensional definition from the World Health Organization (WHO). As far as methodology is concerned, the low correlation coefficient shows that work span is indeed capturing a new structure of aging; specifically, the labor/economic component of aging.

### 3.4 Regression analyses

In order to forecast the scores on healthy aging across different countries and to quantify the importance of the individual constituents of the score, a range of regression models has been used. The models used within this research work range from the conventional methods to the modern approaches based on the Machine Learning algorithm. A list of the models used herein includes Linear Regression, Ridge, Lasso, Elastic Net, Bayesian Ridge, Huber Regressor, Support Vector Regression with Radial Basis Function kernel, k-Nearest Neighbors (k-NN), Gradient Boosting, Random Forest, XGBoost, LightGBM, CatBoost, AdaBoost, Decision Tree, and Multi-Layer Perceptron, aka Artificial Neural Network.

The model performances were measured for Mean Squared Error (MSE), Root Mean Squared Error (RMSE), Mean Absolute Error (MAE), and R^2^ (Coefficient of Determination). The observations suggest that linear models such as Huber, Bayesian Ridge, and traditional Linear Regression performed very well in terms of predictive accuracy for the HAPI components. However, the Tree-based models had a slightly lower R^2^ measure of accuracy, yet they performed very well for discovering the complex non-linear patterns.

The regression results essentially form the basis for interpreting the contribution of the various components in the overall score. Linear models will explain the straightforward relationship between the variables. The complexity will be captured using the ensemble models and the neural networks. The interaction enables the policy makers to jointly analyze the impact that the various components have on the score for healthy aging for the different countries (22).

In order to assess the relationship between the healthy ageing index and its determinants, regression models have been used. The general linear regression model can be specified as in Equation 4.


$$\begin{array}{l} y_{i}=\beta_{0}+\overset{p}{\underset{j=1}{\sum }}\beta_{j}X_{ij}+\varepsilon_{i} \end{array}$$ (4)

In Equation 4, *y*_*i*_ is used to represent the HAPI score for country *i*, *X*_*ij*_ is used for the *j*-th explanatory variable for the country *i*, β_*j*_ represents regression coefficients for each variable, and ε_*i*_ represents a stochastic error term for each country. However, techniques like Ridge and LASSO regularization aim to reduce overfitting and enable a model to generalize (23).

### 3.5 K-Means clustering analysis

K-Means clustering analysis was then conducted, where countries were grouped according to composite scores derived from the outputs of PCA and regression results. The K-Means algorithm is an unsupervised machine learning algorithm that aims at partitioning observations into *K* non-overlapping clusters by minimizing within-cluster variance. Hence, this algorithm groups countries with similar healthy aging and welfare profiles and highlights the existence of systematic differences. This clearly serves useful purposes in policy analysis, since clusters can be identified that may require differentiated interventions.

The K-Means algorithm aims to minimize the within-cluster sum of squares (WCSS) and is expressed mathematically as in Equation 5 (24–32).


$$\begin{array}{l} WCSS=\overset{K}{\underset{i=1}{\sum }}\underset{\text{x}\in C_{i}}{\sum }||\text{x}-\mu_{i}|{|}^{2} \end{array}$$ (5)

In Equation 5, *C*_*i*_ represents the set of observations in the *i*-th cluster, **x** represents an observation, and **μ**_*i*_ represents the cluster center. The cluster centers are updated iteratively as in Equation 6.


$$\begin{array}{l} \mu_{i}=\frac{1}{\left|C_{i}\right|}\underset{\text{x}\in C_{i}}{\sum }\text{x} \end{array}$$ (6)

In this study, Euclidean Distance, Manhattan Distance, Cosine Similarity, and Correlation Similarity measures were used to evaluate cluster similarities and distances quantitatively.

- *Euclidean distance*: shows the straight-line distance between two set centers. Mathematically, the distance between the two cluster centers **μ**_**i**_ and **μ**_**j**_ is calculated as in Equation 7 (33–35).

$$\begin{array}{l} d_{ij}^{Euclidean}=\sqrt{\overset{p}{\underset{p=1}{\sum }}{\left(\mu_{i,p}-\mu_{j,p}\right)}^{2}} \end{array}$$ (7)

In Equation 7, *P* represents the number of dimensions, and μ_*i, p*_ represents the p-th dimension of the center of the i-th set.

- *Manhattan distance*: it gives the sum of the absolute differences in all dimensions. Mathematically, it is calculated as in Equation 8 (36–39).

$$\begin{array}{l} d_{ij}^{Manhattan}=\overset{p}{\underset{p=1}{\sum }}|\mu_{i,p}-\mu_{j,p}| \end{array}$$ (8)

- *Cosine similarity*: directional similarity is found by calculating the cosine of the angles between the centers of the clusters. Cosine Similarity is calculated using the mathematical formulation in Equation 9 (40–43).

$$\begin{array}{l} CosineSim_{ij}=\frac{\mu_{i}\cdot \mu_{j}}{∥\mu_{i}∥∥\mu_{j}∥} \end{array}$$ (9)

In Equation 9, represents the dot product, and ∥**μ**_*i*_∥ represents the magnitude of the vector.

- *Correlation similarity*: the linear relationship between cluster centers is measured by Pearson correlation (44–47). Correlation Similarity is calculated using the formulation in Equation 10.

$$\begin{array}{l} CorrSim_{ij}=\frac{Cov\left(\mu_{i},\mu_{j}\right)}{\sigma_{\mu_{i}}\sigma_{\mu_{j}}} \end{array}$$ (10)

In Equation 10, *Cov*(**μ**_*i*_, **μ**_*j*_) denotes the covariance computed across the feature dimensions between the component-wise values of cluster center vectors *i* and *j*. The terms σ_**μ**_*i*__ and σ_**μ**_*j*__ represent the standard deviations of the corresponding cluster center vectors across the same dimensions.

### 3.6 SHAP analysis

To understand the contribution of each of the HAPI components to the predicted healthy aging score, the SHAP method was used. SHAP is a XAI framework based on cooperative game theory, which aims to explain the prediction of a machine learning model by its individual input features in a mathematically consistent way (48).

SHAP is based on the Shapley value concept, which was introduced in game theory as a way of distributing the payoff of a coalition among its members according to their marginal contribution (49). The “players” in a machine learning model correspond to its individual features, and the “payoff” is its prediction.

Let **x** represent the vector of **p** explanatory variables and be defined as in Equation 11.


$$\begin{array}{l} \text{x}=\left(x_{1},x_{2},...,x_{p}\right) \end{array}$$ (11)

The **x** vector represents the explanatory variables corresponding to the HAPI indicators. These explanatory variables can be expressed as follows.

- *x*_1_= life expectancy
- *x*_2_= healthy life expectancy
- *x*_3_= working life span
- *x*_4_ = income level
- *x*_5_ = environmental performance
- *x*_6_ = subjective happiness score

Also, let's denote the predictive model used to estimate the healthy aging score as *f*(**x**). For a given country *i*, the predicted value can be expressed as in Equation 12.


$$\begin{array}{l} y_{i}=f\left(x_{i1},x_{i2},...,x_{ip}\right) \end{array}$$ (12)

The SHAP model decomposes the prediction into additive contributions from each feature, following the additive explanation model proposed by Lundberg and Lee (48), as expressed in Equation 13.


$$\begin{array}{l} f\left(\text{x}\right)=\phi_{0}+\overset{p}{\underset{j=1}{\sum }}\phi_{j} \end{array}$$ (13)

The terms *d* and *w* in the equation can be expressed as follows, respectively.

- ϕ_0_ = *E*[*f*(*X*)] represents the expected model prediction across the dataset
- ϕ_*j*_ denotes the contribution of feature *j* to the prediction.

This allows the prediction of the complex machine learning model to be represented as the sum of the feature-level effects.

The SHAP value ϕ_*j*_ for feature *j* is determined using the Shapley value formulation based on cooperative games. The Shapley value represents the average marginal contribution of the feature over all possible combinations of features. It is based on the work of Shapley (49).

The Shapley value for feature *j* is represented as in Equation 14 (48).


$$\begin{array}{l} \phi_{j}=\underset{S\subseteq F\backslash \left\{j\right\}}{\sum }\frac{|S|!\left(p-|S|-1\right)!}{p!}\left[f_{S\cup \left\{j\right\}}\left(x_{S\cup \left\{j\right\}}\right)-f_{S}\left(x_{S}\right)\right] \end{array}$$ (14)

The following expressions can be written for Equation 14.

- *F* denotes the set of all features
- *S* represents a subset of features not containing *j*
- ∣*S*∣ denotes the cardinality of subset *S*
- *p* denotes the total number of features.

The weighting factor


$$\begin{array}{l} \frac{|S|!\left(p-|S|-1\right)!}{p!} \end{array}$$ (15)

ensures that the average value of the marginal contribution of each feature is computed over all possible permutations of feature values. This satisfies the properties of fairness, efficiency, and symmetry that are ensured by additivity (48).

In real-world applications of machine learning, SHAP value calculation is performed by using the conditional expectation representation. The expected prediction is defined as in Equation 16.


$$\begin{array}{l} f_{S}\left(x_{S}\right)=E\left[f\left(X\right)|X_{S}=x_{S}\right] \end{array}$$ (16)

In Equation 16, *X*_*S*_ represents the subset of known variables.

Accordingly, the SHAP value can be interpreted as the expected change in the model prediction when feature *j* is added to subset *S*:


$$\begin{array}{l} \phi_{j}=E_{S\subseteq F\backslash \left\{j\right\}}\left[f\left(x_{S\cup \left\{j\right\}}\right)-f\left(x_{S}\right)\right] \end{array}$$ (17)

This definition enables SHAP to offer consistent explanations for various machine learning models, including linear models, tree-based models, and neural networks (48, 50).

In order to assess the overall influence of each of the HAPI indicators across all countries, the global feature importance was computed by using the mean absolute SHAP value. This is defined in Equation 18.


$$\begin{array}{l} I_{j}=\frac{1}{N}\overset{N}{\underset{i=1}{\sum }}|\phi_{ij}| \end{array}$$ (18)

In Equation 18, *N* is the number of observations (countries), and ϕ_*ij*_ is the SHAP value of feature *j* for country *i*. *I*_*j*_ is defined as the average contribution magnitude of feature *j* across the dataset, which is one of the most commonly used measures of variable importance in studies of interpretable machine learning (51, 52)

### 3.7 Policy simulation analyses

Finally, in the last step of the analysis, the policy scenario simulations were performed based on the results of the regression models, PCA composite scores, and SHAP values calculated in the preceding steps. At this step of the analysis, the aim is to estimate the potential effect of changes in the values of certain HAPI indicators on the global healthy ageing score at the country level. Techniques of policy simulations have been frequently applied in health economics and public policy research in the assessment of the potential effect of policy interventions (53, 54).

Let the global healthy ageing score for the country *i* be represented as a function of the HAPI indicators in the form of Equation 19.


$$\begin{array}{l} HAPI_{i}=f\left(L_{i},HL_{i},W_{i},I_{i},E_{i},H_{i}\right) \end{array}$$ (19)

In Equation 19, *L*_*i*_ stands for life expectancy, *HL*_*i*_ stands for healthy life expectancy, *W*_*i*_ stands for working life span, *I*_*i*_ stands for income level, *E*_*i*_ stands for environmental performance, *H*_*i*_ stands for subjective happiness.

This functional form is a reflection of the multi-dimensional nature of healthy ageing indicators, as well as composite indices commonly employed in social policy analysis (21).

The regression model obtained in the previous stage is an empirical form of this function in Equation 12.


$$\begin{array}{l} HAPI_{i}=\beta_{0}+\overset{p}{\underset{j=1}{\sum }}\beta_{j}X_{ij}+\varepsilon_{i} \end{array}$$ (20)

Her *X*_*ij*_ in Equation 20 represents the *j*-th component of HAPI for the *i*-th country, β_*j*_ is the marginal impact of the *j*-th component, and ε_*i*_ is the error term. The above linear regression model is commonly employed in econometric modelling for the estimation of the marginal impact of the independent variable on the dependent variable (55).

To illustrate the potential of various policy interventions, hypothetical improvements in certain indicators are postulated. Let Equation 21 below represent the improved value of indicator *j*, where Δ_*j*_ represents the simulated policy change.


$$\begin{array}{l} X_{ij}^{*}=X_{ij}+\Delta_{j} \end{array}$$ (21)

The resulting predicted healthy ageing score is as shown in Equation 22.


$$\begin{array}{l} {\hat{HAPI}}_{i}^{*}=\beta_{0}+\overset{p}{\underset{j=1}{\sum }}\beta_{j}X_{ij}^{*} \end{array}$$ (22)

Therefore, the estimated policy effect is calculated as in Equation 23.


$$\begin{array}{l} Impact_{i}={\hat{HAPI}}_{i}^{*}-{\hat{HAPI}}_{i} \end{array}$$ (23)

This approach allows for the simulation of the potential impacts of positive change in the results for healthy ageing that could be achieved through policy intervention in particular domains such as environmental performance, income, or labor participation. Simulation-based policy analysis is commonly applied in health policy modeling for the estimation of the impacts of hypothetical policy interventions before their implementation (53, 54).

To include feature importance based on explainable AI techniques, SHAP contribution values are used for weighting. The SHAP contribution values have their basis in the concept of the Shapley value for cooperative games. The SHAP value offers a rigorous approach to interpreting machine learning models (48, 49).

Let ϕ_*j*_ denote the SHAP contribution of feature *j*. The weighted policy impact can be represented by Equation 24.


$$\begin{array}{l} Impact_{i}^{SHAP}=\overset{p}{\underset{j=1}{\sum }}\phi_{j}\Delta_{j} \end{array}$$ (24)

In Equation 24, the formulation allows the simulation framework to not only consider regression coefficients but also the relative importance of each variable based on the machine learning model. SHAP is commonly used in explaining predictive models, particularly in understanding the contribution of each variable to the predictive model (48, 56).

### 3.8 Integrative analysis methodology: Entropy, PCA, Lorenz, clustering, and SHAP

For this research, the integrated assessment of global welfare is carried out using the HAPI index via entropy weighting, PCA-based methods, inequality measures, clustering, and feature importance.

*Data normalization:* the features of HAPI, i.e., *X*_*j*_, were normalized within the range [0, 1] using the min-max normalization technique (18). The mathematical calculation of normalization is shown in Equation 24.


$$\begin{array}{l} X_{ij}^{norm}=\frac{X_{ij}-\min\left(X_{j}\right)}{\max\left(X_{j}\right)-\min\left(X_{j}\right)} \end{array}$$ (25)

*Entropy weight method:* the relative importance of each dimension of HAPI is calculated using the entropy weight *w*_*j*_ of the feature *X*_*j*_, as shown in (57). The entropy weight is calculated using Equation 25.


$$\begin{array}{l} P_{ij}=\frac{X_{ij}^{norm}}{\overset{n}{\underset{i=1}{\sum }}X_{ij}^{norm}},e_{j}=-k\overset{n}{\underset{i=1}{\sum }}P_{ij}\ln\left(P_{ij}\right),w_{j}=\frac{1-e_{j}}{\overset{p}{\underset{j=1}{\sum }}\left(1-e_{j}\right)} \end{array}$$ (26)

In Equation 26 *n* is the number of countries, *p* is the number of features, and *k* = 1/*ln*(*n*). The entropy-weighted HAPI score for country *i* is calculated as in Equation 27.


$$\begin{array}{l} HAPI_{i}^{entropy}=\overset{p}{\underset{j=1}{\sum }}w_{j}X_{ij}^{norm} \end{array}$$ (27)

**PCA-based index:** an alternative HAPI score was created using a weighted PCA combination of features:


$$\begin{array}{l} HAPI_{i}^{PCA}=\overset{p}{\underset{j=1}{\sum }}\alpha_{j}X_{ij} \end{array}$$ (28)

In Equation 28 α_*j*_ are PCA-derived weights reflecting the variance explained by each component.

*Inequality assessment:* the distribution of welfare was assessed using the Lorenz curve and the Gini coefficient (57).


$$\begin{array}{l} G=\frac{n+1-2\overset{n}{\underset{i=1}{\sum }}cum_{i}}{n} \end{array}$$ (29)

In Equation 29 *cum*_*i*_ is the cumulative sum of sorted $HAPI_{i}^{entropy}$.

Feature importance (SHAP): the HAPI feature contributions to the entropy-based index were measured using SHAP values (ϕ_*j*_), which represent additive contributions to the prediction made by the model (48, 49):


$$\begin{array}{l} HAPI_{i}^{entropy}=\phi_{0}+\overset{p}{\underset{j=1}{\sum }}\phi_{j} \end{array}$$ (30)

The global feature importance is calculated as the average of the absolute values of the SHAP values for all countries:


$$\begin{array}{l} I_{j}=\frac{1}{N}\overset{N}{\underset{i=1}{\sum }}|\phi_{ij}| \end{array}$$ (31)

The above integrated method offers a robust framework for measuring welfare, inequalities between countries, feature contributions, and structural clustering.

## 4 Analysis results and empirical findings

### 4.1 Correlation structure and principal component analysis of HAPI indicators

**Figure 2:**
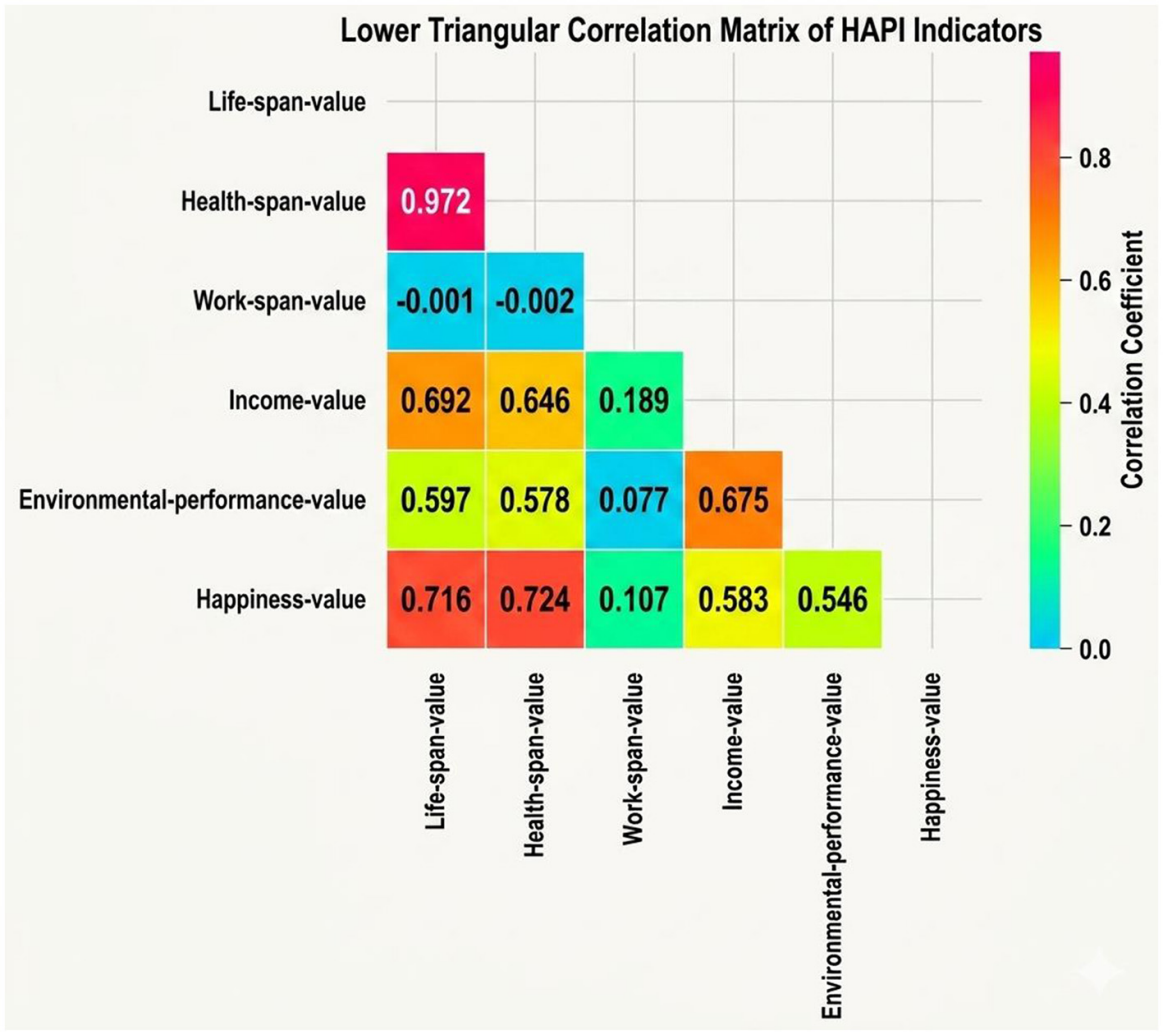
Lower triangular correlation matrix of HAPI indicators based on MICE-imputed data.

Figure 2 displays the linear correlations between the six critical HAPI indicators based on a data set containing imputed missing data using the MICE technique. The lower triangular section of the correlation matrix shows the strength and direction of the relationships between the variables. There is a very high correlation between life-span and health-span, with *r* = 0.972, suggesting that nations with high life expectancy will have high health outcomes as well. Income, environmental performance, and happiness are moderately to strongly correlated with life-span and health-span, with income and happiness showing *r* = 0.692 and *r* = 0.716, respectively, indicating the close relationship between economic and subjective well-being and long and healthy lives. Environmental performance is strongly correlated with life-span and health-span, suggesting that sustainability and healthy aging go hand in hand.

In contrast, the correlations of work-span with the rest of the HAPI indicators are relatively low in magnitude. For example, the correlations with life-span and health-span are almost zero (*r* = 0), and the correlations with income, environmental performance, and happiness are low, suggesting that work-span is an independent variable in the HAPI index. These findings are consistent with the multi-dimensional nature of the HAPI index in the sense that, although life expectancy, health, income, the environment, and happiness are closely related, work-span is distinct and divergent in its relationship with the rest of the variables in the analysis. These findings are consistent with theoretical expectations in a MICE-imputed dataset and provide a solid statistical foundation for the analysis.

Principal component analysis was employed to reduce dimensionality and discern the latent structure underlying six core indicators of the Healthy Ageing and Prevention Index: PCA is an appropriate means through which multidimensional information can be summarized under conditions of substantial conceptual interdependence and empirical correlation among life-span, health-span, work-span, income, environmental performance, and happiness. Rather than imposing either arbitrary or equal weights, PCA derives orthogonal components that maximize explained variance in a statistically rigorous approach that develops an empirically grounded representation of cross-country differences in healthy ageing and human well-being.

**Figure 3:**
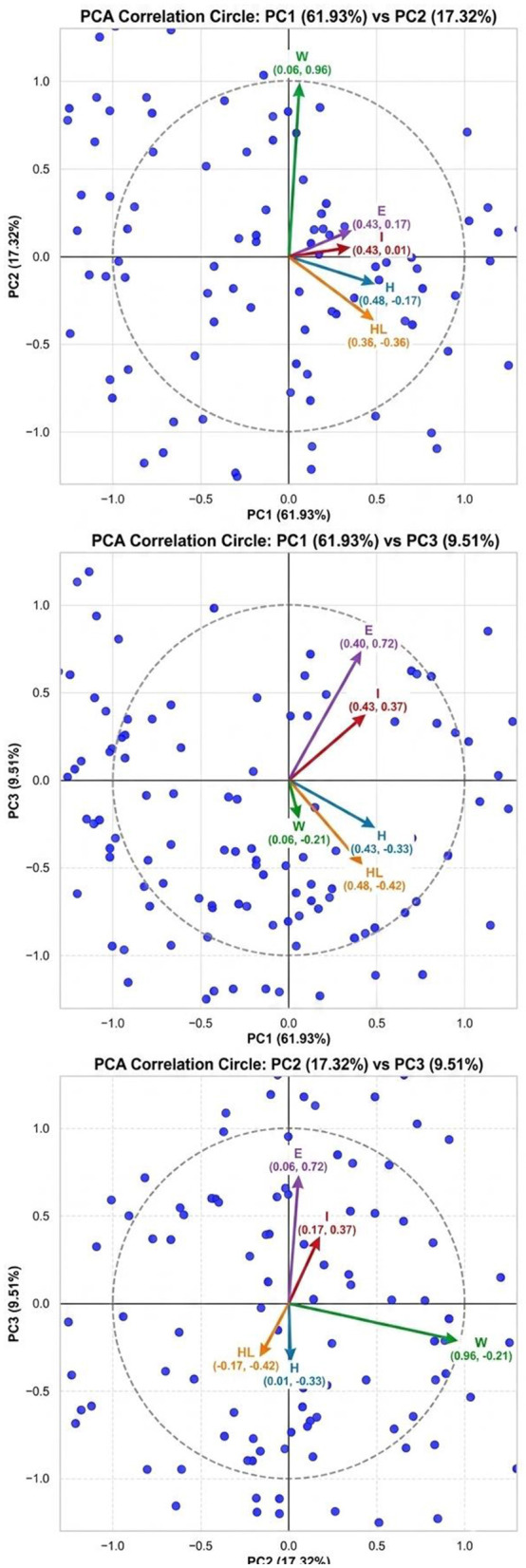
PCA unit circle plots for HAPI indicators (PC1–PC2, PC1–PC3, PC2–PC3).

Figure 3 displays the PCA outcome for the six fundamental HAPI indicators, showing the percentage of variance explained as well as the factor loading of the six HAPI indicators on the first three principal components. The first principal component, PC1, explains 61.93% of the total variance, loading highly on life span (0.483), health span (0.477), income (0.433), environmental performance (0.403), and happiness (0.431). These high positive factor loadings suggest that PC1 represents a holistic concept of general well-being, incorporating life span, health span, income, environmental performance, and happiness into a single construct of sustainable prosperity. As shown in Figure 3, the clustering of the six HAPI indicators along PC1 confirms the notion of a comprehensive measure of sustainable prosperity, where a country with a high score in any of the six HAPI indicators tends to score high in all the other five HAPI indicators as well.

The second and third principal components identify variables that are conceptually different from the construct of well-being in general. PC2, which accounts for an additional 17.32% of the total variance, is largely dominated by the variable of work-span (0.957), with minimal contributions from the rest of the variables. PC3, which accounts for an additional 9.51% of the total variance, is largely associated with the variable of environmental performance (0.719) and moderately with income (0.371), with negative loadings for variables of life-span (−0.278), health-span (−0.338), and happiness (−0.332). It is evident from Figure 3 that PC1 unifies all the variables under a single construct of well-being, whereas PC2 and PC3 identify different structural variations in the data for different countries, thereby validating a parsimonious and multi-dimensional representation of the HAPI index.

The results from the PCA analysis allow for the calculation of a data-driven composite Healthy Aging and Prevention Index (HAPI-PCA), where the weight assigned to each component is endogenously calculated and not arbitrarily set at an equal weight. This allows the relative weight assigned to each component to reflect its explanatory power in explaining the underlying variance across countries, as depicted in Figure 3.

Consistent with the component retention strategy identified in this Section, the first three principal components (PC1, PC2, and PC3), which collectively explained 88.77 percent of the total variance, were retained in the calculation of the composite index. Accordingly, the PCA-based HAPI index for country ‘*i*' can be written as:


$$\begin{array}{l} HAPI_{PCA,i}=w_{1}\cdot PC_{1i}+w_{2}\cdot PC_{2i}+w_{3}\cdot PC_{3i} \end{array}$$ (32)

The weights *w*_1_, *w*_2_ and *w*_3_ Equation 11 corresponds to the ratios of variance explained by the respective components.


$$\begin{array}{l} w_{1}=0,6193,\;\;w_{2}=0,1732,w_{3}=0,0951 \end{array}$$ (33)

The developed composite values are then normalized to a scale of [0–100] for easier interpretability and cross-country comparability. Under this weight scheme, PC1, which represents overall well-being in terms of life span, health span, income, environmental performance, and happiness, contributes the most to the developed HAPI index. PC2 represents labor participation and work span, and PC3 represents environmental economic trade-offs, which contribute proportionally to the developed HAPI index. By using PCA to develop the HAPI index, it is ensured that it represents a holistic and balanced measure of healthy aging, which goes beyond life expectancy or income but incorporates objective and subjective dimensions of human well-being.

### 4.2 Multidimensional clustering, profiling, and classification of HAPI-based country groups

On the basis of the PCA-based HAPI index constructed in Section 4.1, a clustering analysis was carried out with a view to classifying countries with equal healthy aging and well-being indicators. The major aim of this analysis is two-fold: first, to reduce high-dimensional indices from PCA scores to easily interpretable country groups; and, secondly, to highlight differences between countries to serve as a clue for well-informed policymaking practices.

In PCA, the first three principal components (PC1, PC2, and PC3) were used for clustering. This method ensures that in clustering, overall well-being/sustainable development (PC1), integration in the working life (PC2), and environmental/economic stresses (PC3) are taken into account. This helps in avoiding multicollinearity in the indicators.

**Figure 4:**
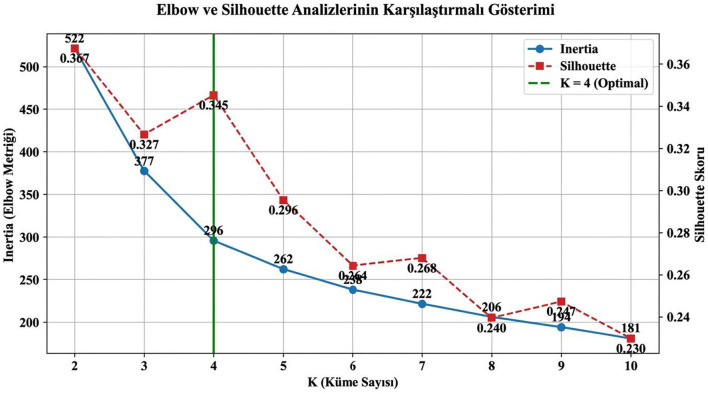
Elbow and Silhouette analysis plots for determining optimal number of clusters.

The Elbow and Silhouette analyses were conducted within the range *K* = 2–10 to identify the choice of *K* representing the optimal number of clusters. As captured in Figure 4, with an increasing *K*, the inertia values decrease while the silhouette score reaches a local maximum at *K* = 4. This points to the fact that *K* = 4 would be an appropriate choice because, for this value, sufficient inter-cluster separation and intra-cluster consistency exist.

**Figure 5:**
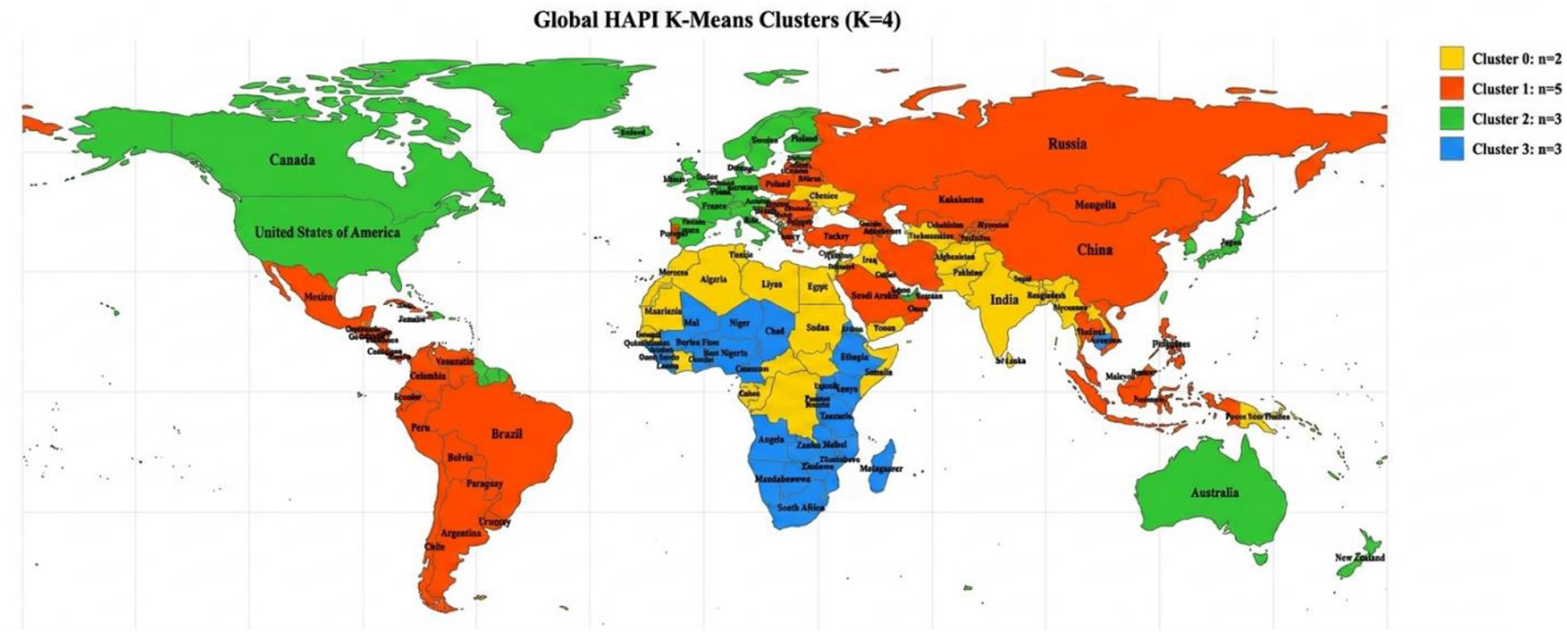
Global HAPI K-Means clusters (K = 4) mapped on world map.

Subsequently, the application of the K-Means algorithm for the clustering process with the value of *K* set to 4 helped visualize the clusters on the global map with the clusters represented in the map depicted in Figure 5.

The four resultant groups from the analysis show different profiles across countries with respect to healthy aging and well-being factors:

Cluster 0 (27 countries): this group is dominated by South Asian, North African, and Middle Eastern nations such as Afghanistan, Bangladesh, Egypt, Iran, and Yemen. These nations usually rank low on PC1 indices and overall happiness, with PC2 and PC3 factors being moderate or low as well. This is attributed to life expectancy, health-related issues, income levels, and overall environmental outcomes.

Cluster 1 (58 countries): this group is quite divergent and includes Latin American, Eastern European, and some Asian countries. These countries include Argentina, Brazil, China, Turkey, and Vietnam. The values for PC1 are moderate, whereas the levels for both PC2 and PC3 differ for each country. This suggests that for different countries, there might be varying factors influencing HAPI.

Cluster 2 (30 countries): comprises Western European affluent countries, North American countries, Oceanian countries, and some countries in East Asia; for example, Australia, Germany, Japan, and USA. The countries belong to those with very high scores in PC1 for overall well-being, health, income, and life satisfaction. The scores for PC2 and PC3 are also positive. It can be referred to as an example for ‘best practices' worldwide.

Cluster 3 (38 countries): mostly comprised of Sub-Saharan African nations like Angola, Ethiopia, Kenya, Nigeria, and Zimbabwe. These countries are relatively deprived in terms of general well-being as well as their level of engagement in working life due to low PC1 and PC2 values but have fluctuating PC3 values reflecting the diversity of structural factors.

**Figure 6:**
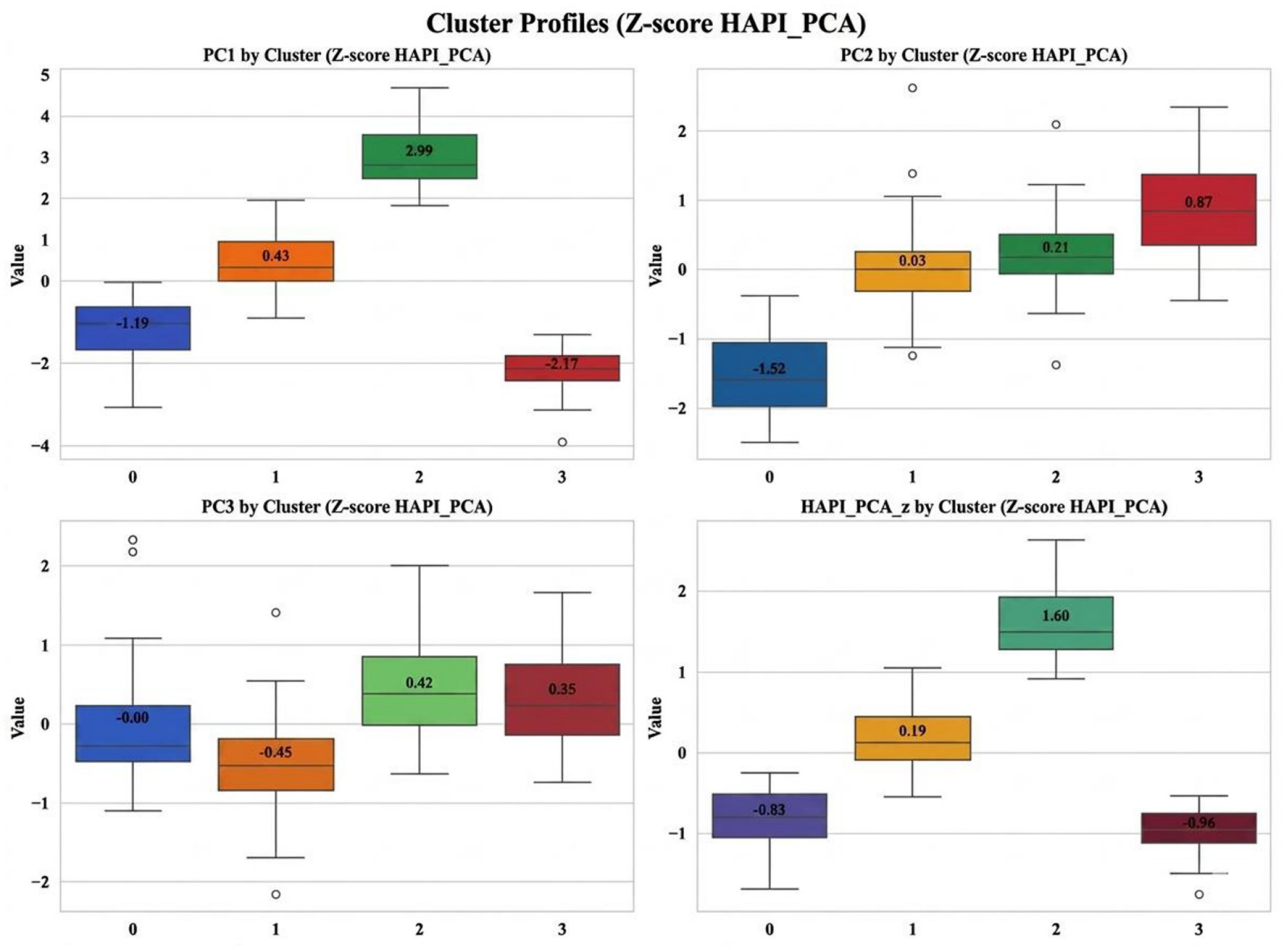
Cluster profiles of countries based on PC1–PC3 and HAPI-PCA scores.

After clustering the data with K-Means clustering on the PCA-based HAPI Index, each of the clusters is examined with regard to PC1, PC2, PC3, and Z-score normalized HAPI_PCA scores. Figure 6 is a boxplot showing all four of these features across all of the clusters. It is evident from this visualization that there are indeed numerical variations across each of the clusters. For Cluster 0, countries have low PC1 scores at −1.19 and PC2 scores at −1.52, with a mean HAPI_PCA_z of −0.83. There are major problems with regard to well-being and engagement with the labor force. For Cluster 1, countries have moderate PC1 scores at 0.43 and HAPI_PCA_z at 0.19, indicating average performance with regard to health, well-being, and engagement with the labor force.

Cluster 2 is marked by high values of PC1 (2.99) and HAPI_PCA_z (1.60), indicating countries that perform extremely well in terms of well-being, health, income levels, and overall happiness levels. These countries are mostly in Western Europe, North America, and Oceania, as shown in the boxplots. On the other hand, Cluster 3 is marked by extremely low values of PC1 (−2.17) but moderate to high values of PC2 (0.87) and low values of HAPI_PCA_z (−0.96), indicating disadvantaged countries that are moderately engaged in the labor force but have low levels of overall well-being (Figure 6).

The boxplots clearly show that not only do the clusters differ nominally but also numerically. This is a crucial finding for policy and practice because it shows that for Cluster 0 and Cluster 3, interventions in health, education, and environmental areas are necessary, while Cluster 2 is an example of countries that perform well in all areas. This analysis shows that PCA clustering is successful in capturing multidimensional differences in healthy aging and well-being, as measured by Z-score normalized HAPI_PCA values.

**Figure 7:**
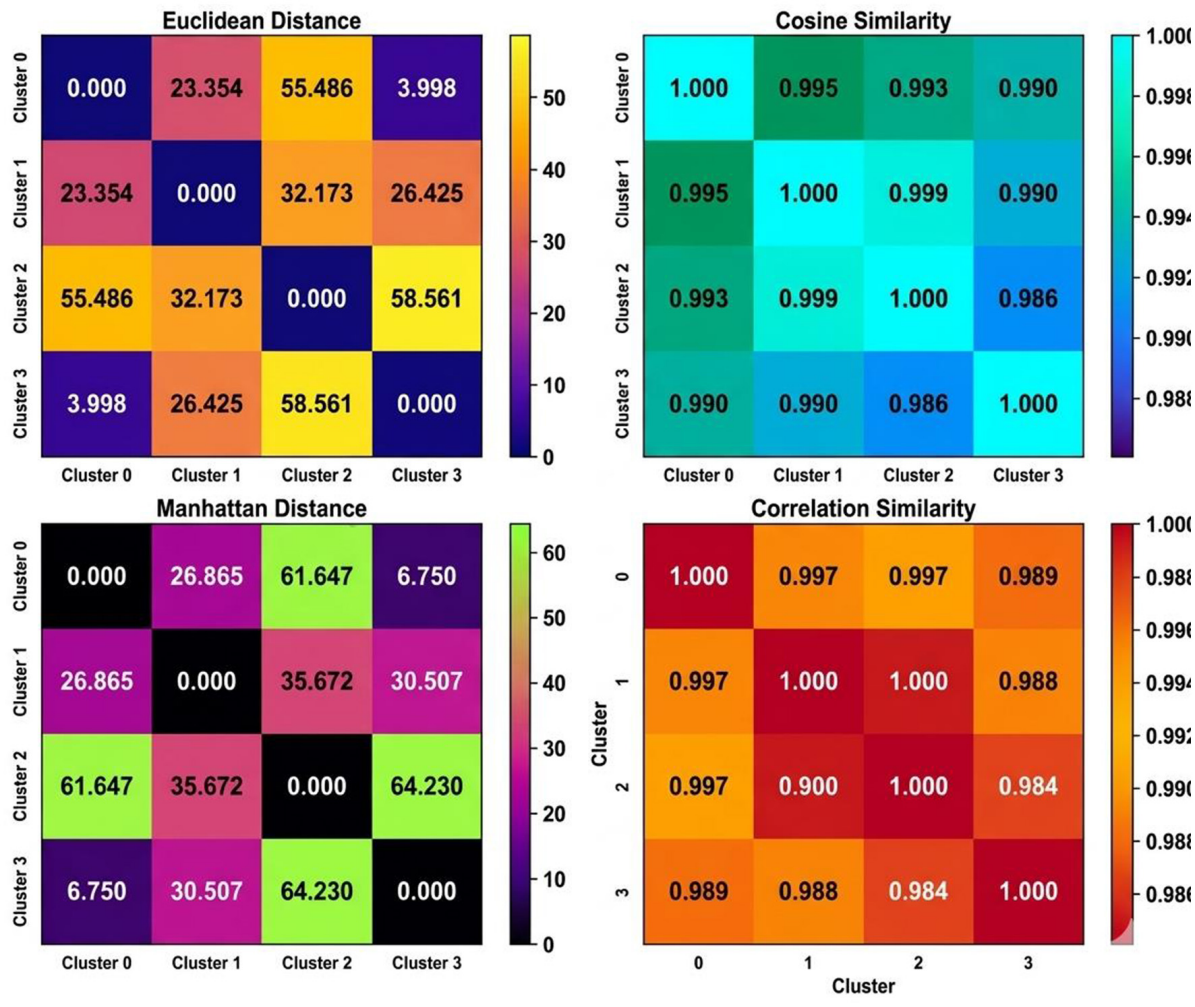
Cluster similarity matrices (Euclidean, Cosine, Manhattan, Correlation).

Having established the existence of four different country groups based on the results of the PCA-based HAPI scores (refer to Figure 5), the cluster similarity analysis was performed based on the Euclidean, Manhattan, cosine, and correlation metrics to evaluate the absolute, directional, and linear similarities between the clusters with respect to the PC1, PC2, PC3, and HAPI_PCA values. The results of the similarity analysis are given in Figure 7.

The results, as shown in Figure 7, indicate the following important observations: Cluster 2 appears to be the most differentiated, with the highest differences compared to the other clusters, as indicated by the Euclidean and Manhattan distances, which reach up to 58.56 and 64.23, respectively, because of the exceptionally high PC1 and HAPI_PCA values. Cosine similarity and correlation also point to the uniqueness of Cluster 2, although the direction of the alignment remains high because of the similarities in the PC2 and PC3 profiles. Cluster 3 appears to be very close to Cluster 0, as indicated by the high similarities in the Euclidean (3.99) and Manhattan (6.75) distances, yet the correlation similarity is low (0.9891), which indicates the structural differences in the two profiles, despite the high proximity. Cluster 1 appears to be at an intermediate position, being moderately similar to both Cluster 0 and Cluster 2, which indicates the heterogeneous nature of the characteristics of Cluster 1 compared to the low and high performance clusters.

**Figure 11:**
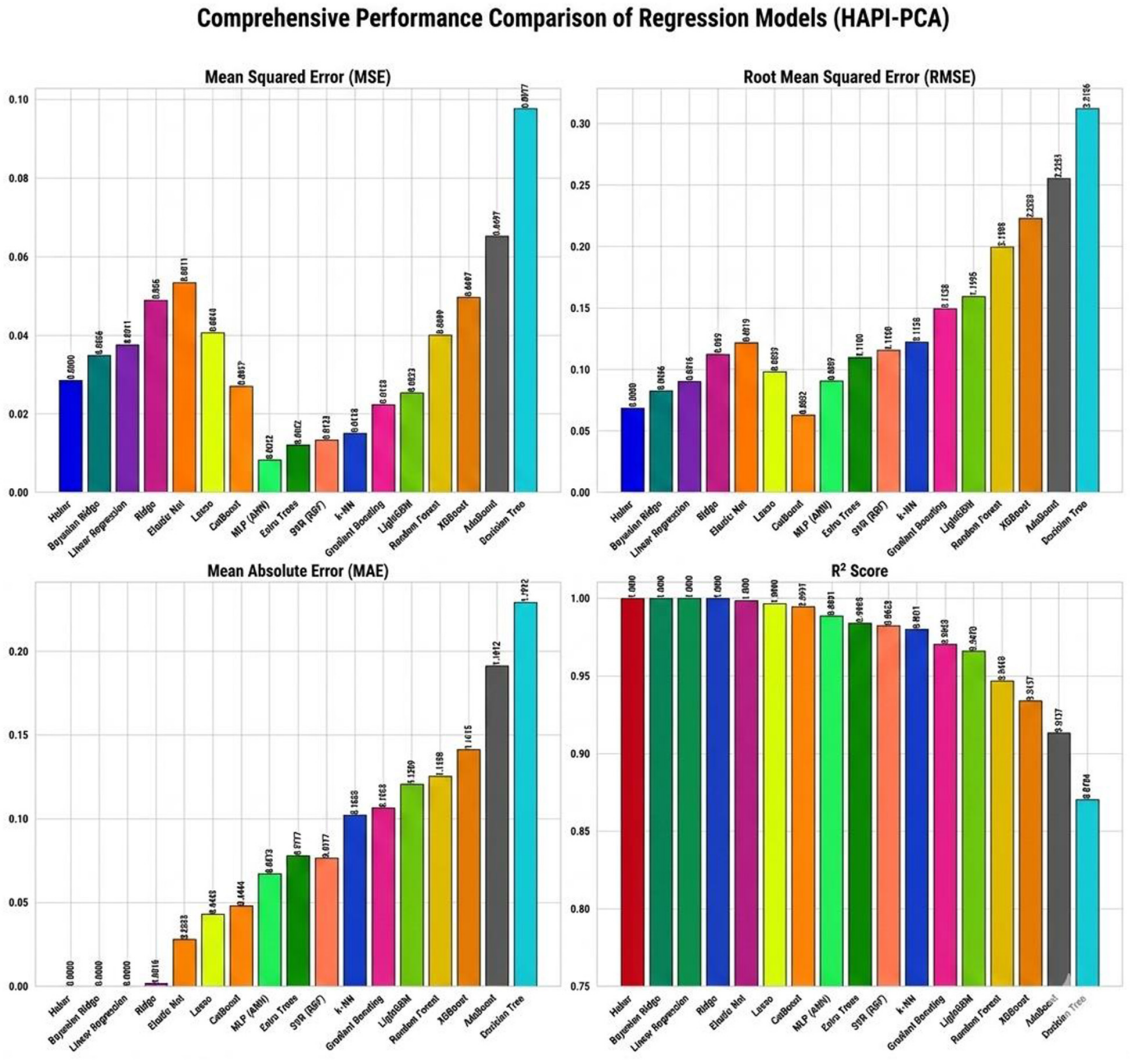
Performance metrics of final linear regression models.

Thus, it is observed that the quantitative similarity matrices reinforce the earlier observation made from Figure 11, where it was noted that high HAPI_PCA_score clusters (Cluster 2) are well differentiated from low HAPI_PCA_score clusters (Cluster 0 and 3), and Cluster 1 has a mixed profile. This again confirms that not only are the four clusters visually distinct, they are also quantitatively distinct, thereby yielding a robust understanding of global patterns of healthy aging and well-being.

**Figure 8:**
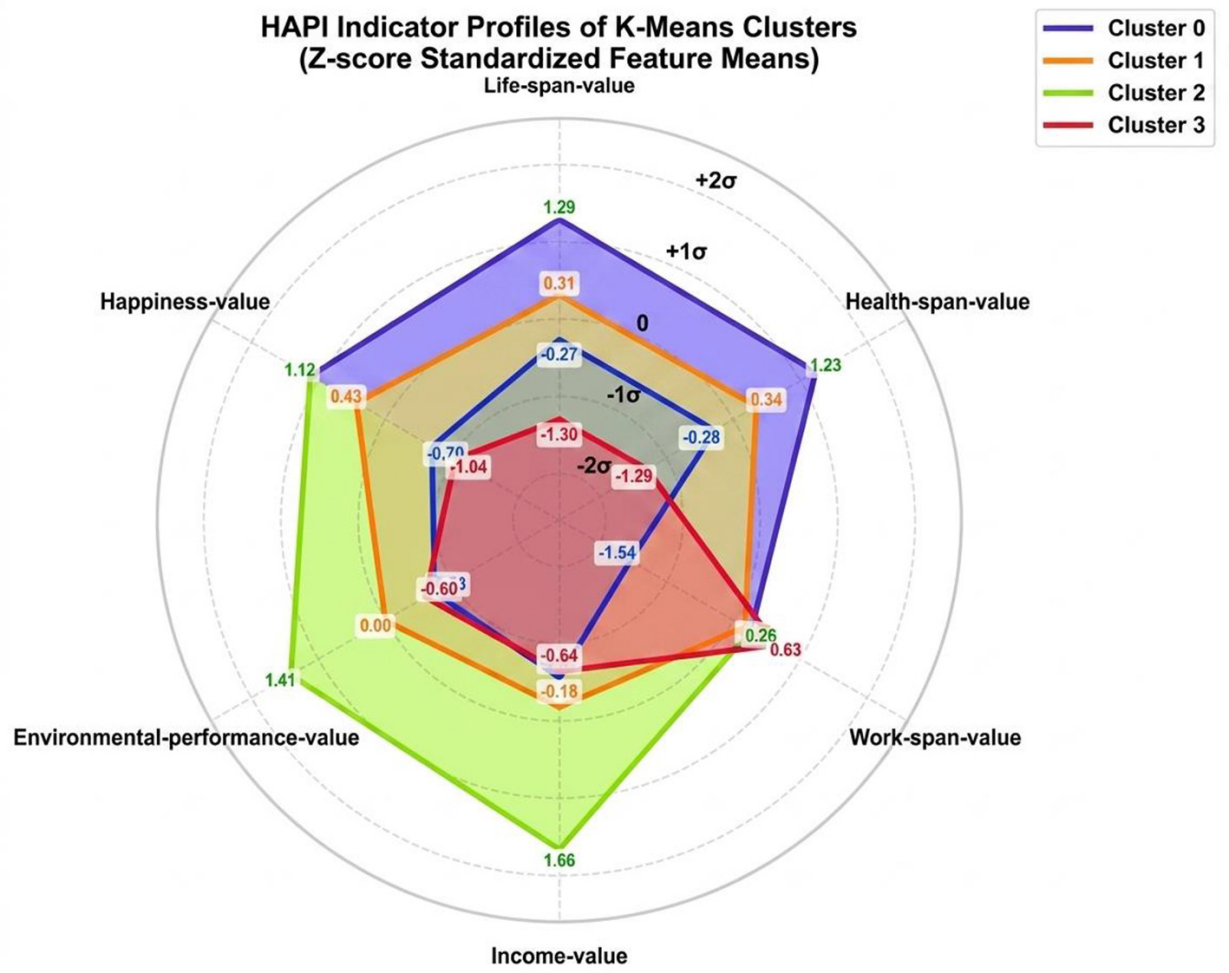
Radar chart of HAPI indicator profiles across K-Means clusters.

In addition to further exploring the structural properties of the obtained results for the developed clusters, the use of radar chart (spider chart) visualization has been considered. The main purpose of the analysis with the use of radar charts is to compare the multidimensional profiles of the developed clusters based on the original HAPI indicators, rather than their reduced forms obtained with the use of the PCA method. By comparing the standardized Z-score means of the life span, health span, work span, income, environmental performance, and happiness indicators for each cluster, the analysis provides an intuitive understanding of the multidimensional differences between the developed clusters. The results of the multidimensional cluster profiling analysis are shown in Figure 8.

Figure 8 depicts the multidimensional differences between the four clusters formed by PCA-K-Means. Cluster 2 consists of highly developed countries with high incomes (+1.66), environmental performance (+1.41), life span (+1.29), health span (+1.23), and happiness (+1.12). Cluster 1 contains moderately performing and structurally stable countries with no attributes above ±1.

Cluster 0 is characterized by low work spans (−1.54) and moderately low life spans (−0.85), incomes (−0.64), environmental performance (−0.55), and happiness (−0.48). Cluster 3 is distinguished by low life spans (−1.30), low health spans (−1.29), low happiness levels (−1.04), and moderately longer work spans (+0.63). The radar chart shows that healthy ageing outcomes depend on complex interactions between various attributes and not merely differences in average indices.

**Figure 9:**
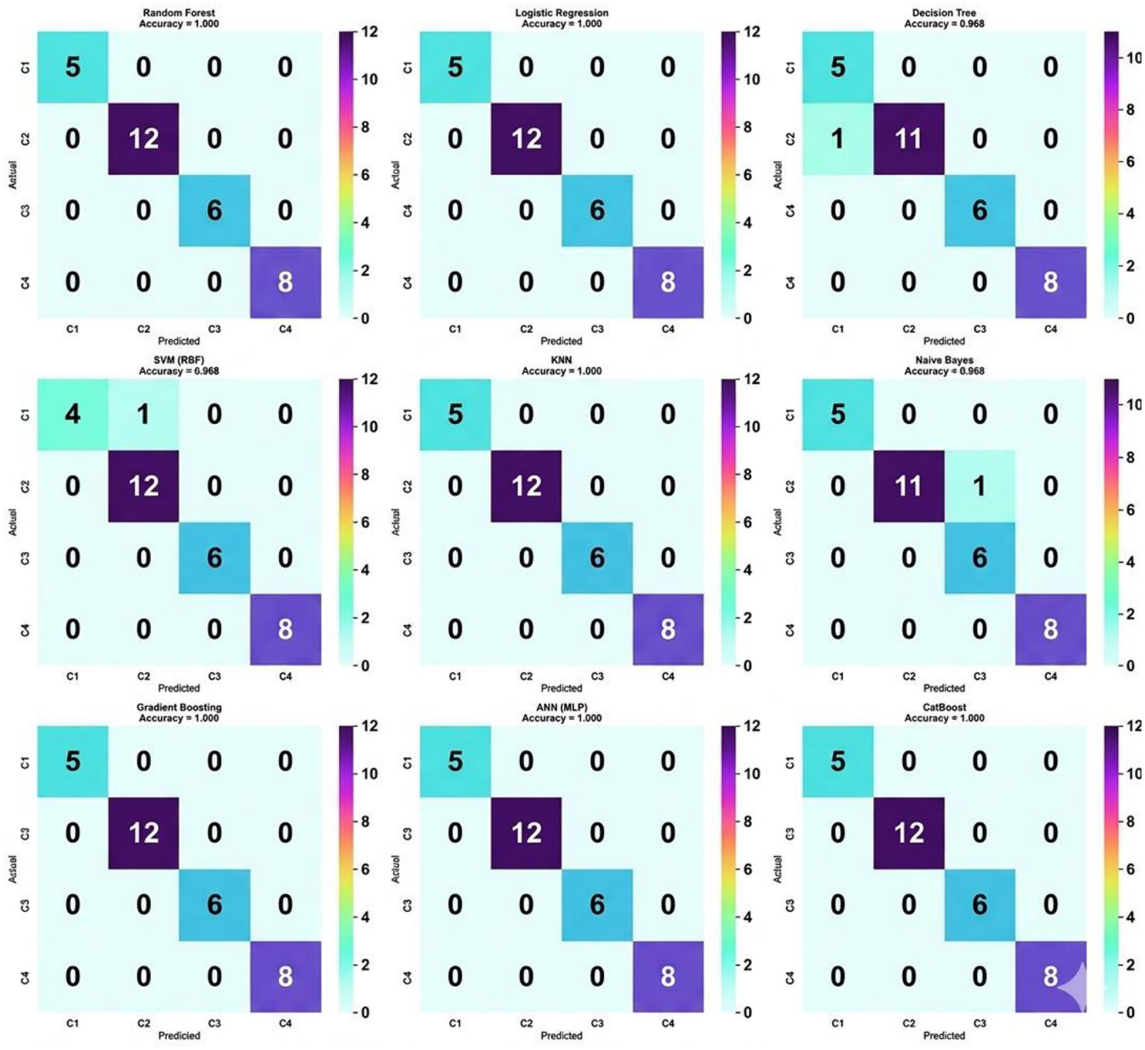
Confusion matrices of machine learning models for predicting HAPI clusters.

As a measure of how distinguishable the clusters that were formed via the PCA-K-Means unsupervised clustering technique were, another supervised classification experiment using machine learning was done. This experiment was designed to test whether different machine learning techniques could actually predict the labels of the cluster using PCA values for HAPI indicators. This is yet another form of validation to determine whether the clusters that were formed by the algorithm are indeed distinguishable. Different machine learning algorithms' classification ability is shown in Figure 9.

performance for the K-Means clusters. Random Forest, Logistic Regression, KNN, ANN, and CatBoost achieved 100% accuracy, implying that the data were clearly distinguishable in the multidimensional feature space. Decision Tree, SVM, and Naïve Bayes achieved slightly lower accuracy (~96.8%), with errors being rare and confined to clusters with partially overlapping attributes.

This demonstrates that PCA-K-Means clustering is not only statistically valid but also highly predictable in its outcomes, implying strong structural separability between country groups. Overall, it may be concluded that HAPI indicators offer a well-structured multidimensional representation of healthy ageing patterns.

**Figure 10:**
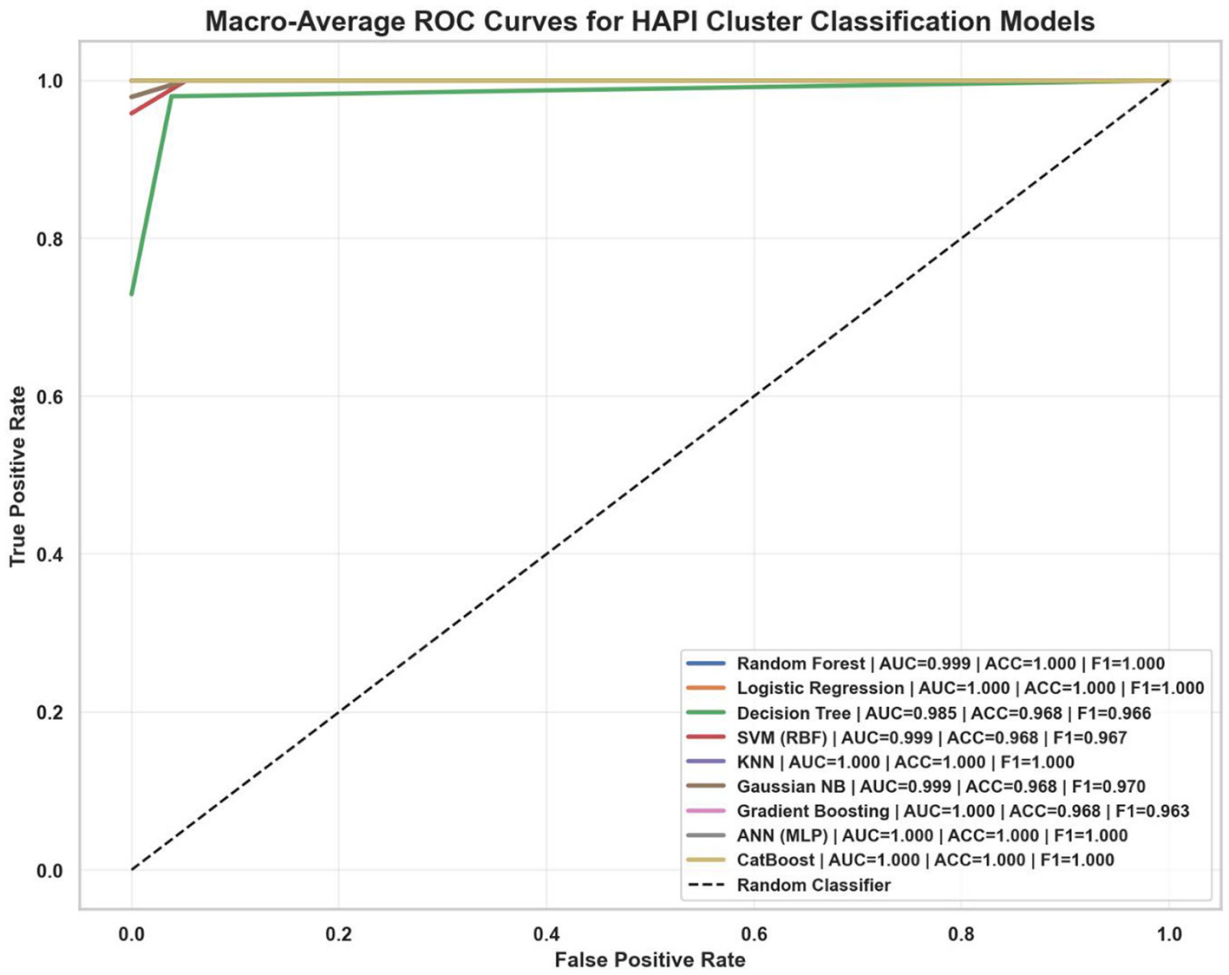
Macro-average ROC curves for HAPI cluster classification models.

To further evaluate the performance of the machine learning models applied in the classification process, Receiver Operating Characteristic (ROC) analysis was applied. ROC curves are commonly used in the analysis of various machine learning and artificial intelligence models. The ROC curve analysis helps evaluate the performance of the models with different threshold levels. For multiclass classification problems, macro-average ROC curve analysis helps evaluate the performance of the models overall, considering the performance of the models on each class. Figure 10 presents the results of the ROC curve analysis.

The ROC curve in Figure 10 above indicates high performance in terms of classification for all the algorithms. The Random Forest, Logistic Regression, KNN, ANN, and CatBoost algorithms all performed well in terms of macro-average AUC, which is close to 1. This is an indication that there is a distinct separation between the four cluster categories.

Even less accurate algorithms like Decision Trees and SVMs have AUC values of more than 0.98. This is a further indication that there is a high level of separation between the clusters in the feature space. The high performance of all algorithms in terms of AUC, accuracy, and precision-recall is an indication that PCA-K-Means is effective in identifying distinct cluster boundaries.

The above analysis is an indication that the HAPI analytical framework is effective in capturing the structural patterns of global healthy ageing trends. The integration of dimensionality reduction, clustering, and classification is a robust approach for conducting comparative cross-country health analysis.

### 4.3 Linear regression models and feature importance analysis

In this section, the linear regression models that presented the best performance, as shown by the results of analysis, are critically discussed. The models include Linear Regression, Ridge Regression, Bayesian Ridge, and Huber Regression. The key reason for this discussion is informed by the performance results demonstrated by Figure 11 below. The four models were able to predict the HAPI-PCA target variable with negligible error, with the explained variance ratio close to 1 (Linear Regression: *R*^2^ = 1.00; Ridge Regression: *R*^2^ = 1.00; Bayesian Ridge: *R*^2^ = 1.00; Huber Regression: *R*^2^ = 1.00). At the same time, the error measurements were also negligible, with values of RMSE and MAE practically zeroing out as shown in Figure 11.

In any case, it should be especially noted that this outcome can be predicted purely from a methodological point of view. The target variable for HAPI-PCA is, by definition, a compound index constructed from linear components that are derived from a linear combination of base indicators. Thus, the statistically correct outcome can only be that linear models, which test their hypotheses within linear parameters, approximate such an index with a high degree of accuracy as compared to any other model. On the other hand, the comparative lack of success of Tree-based models, such as Random Forest, which specifically try to identify the structure of non-linear relationships, as shown by this analysis, is simply because such models strictly clash with the linear data structure.

**Figure 12:**
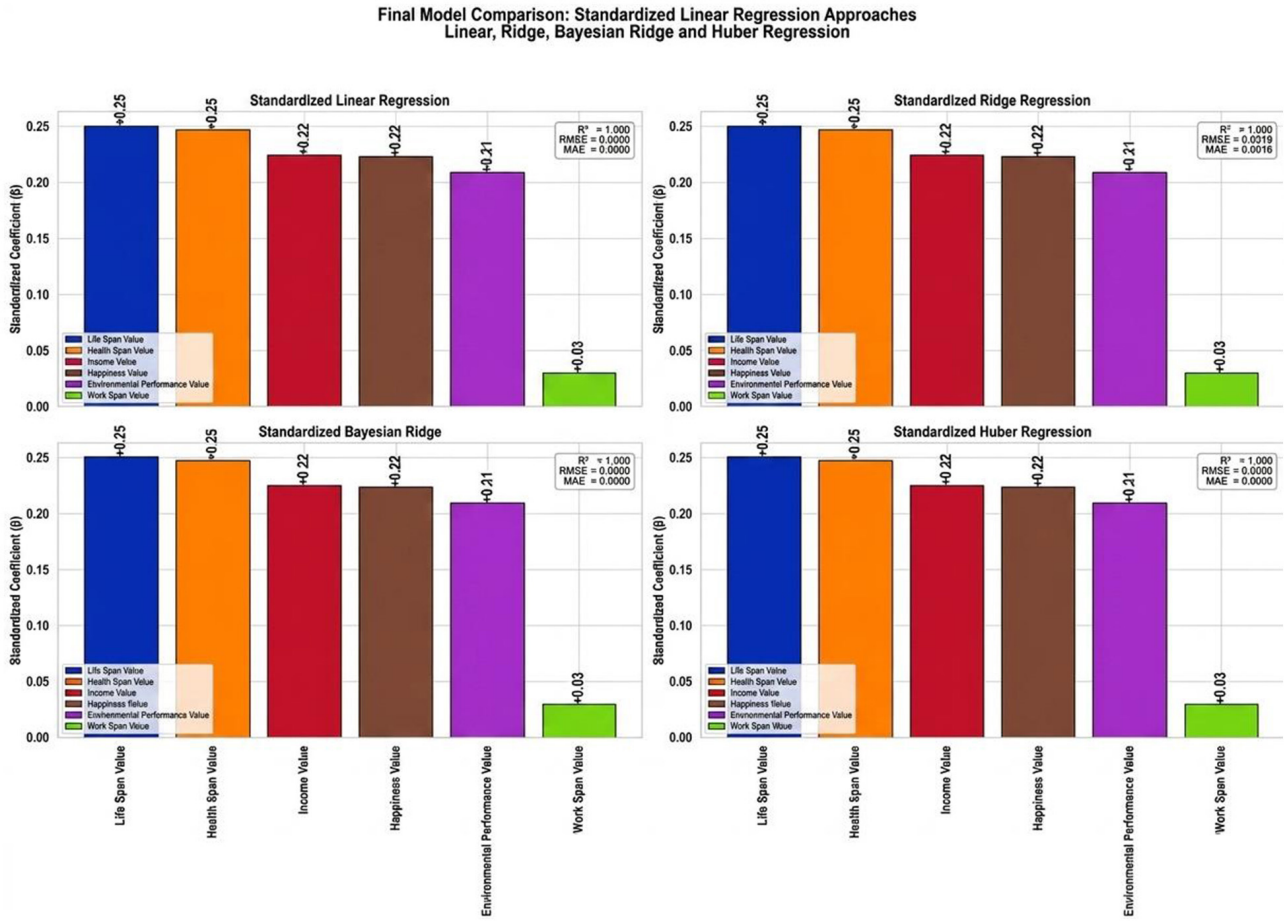
Standardized feature coefficients of linear regression models.

The objective of this analysis step was not only to compare the predictive power of the models but also to disclose the relative importance of the main indicators that make up HAPI-PCA. All independent variables were standardized, as was the target variable of HAPI-PCA; this way, the effects that could arise because of different scales were avoided, and direct comparability of coefficients became possible. Panel graphs in Figure 12 visually present the standardized beta coefficients (β) obtained for each linear model. Results indicate that in all four applied linear models, a highly similar coefficient structure was developed, providing strong support for the hypothesis that the results reflect data structure itself and not a coincidence based on the applied model.

On analyzing the values of standardized coefficients, it has been identified that the variables having maximum influence on the HAPI-PCA are Life-span Value (β = 0.2506) and Health-span Value (β = 0.2472). There is a visible indication of the pivotal role played by the indicators associated with lifespan and health in defining the value of the overall measure of happiness and overall well-being of nations. Income Value (β = 0.2247) and Happiness Value (β = 0.2235) also demonstrate their influential and pivotal role in defining the measure of HAPI-PCA, though the level of influence is slightly lagging behind the role played by the indicators associated with health and lifespan of a nation. Environmental Performance Value demonstrates a moderate influence (β = 0.2093) on the measure, reflecting its auxiliary and supporting role in defining the overall measure of happiness and the overall well-being component. On the contrary, Work-span Value demonstrates a relatively modest influence (β = 0.0301) on the measure of overall happiness and overall well-being of a nation through its associated indicators and structure, indicating the relatively subordinate role played by the working life indicators in comparison with other structural indicators of happiness and overall well-being.

In general, based on the near optimal results presented in Figure 11 and the high degree of coefficient consistency presented in Figure 12, it is safe to conclude that the dominance of Linear Regression Models in this study reflects both theoretically perceived and practically ascertained observations in its entirety, thereby reiterating its relevance in providing optimal results about predictability and interpretability in presenting composite indices like HAPI-PCA and hence, forming the most appropriate analytical perspective for policy analysis and structural inference.

As made evident through the comparative analyses in the former section, the linear regression models forecasted the target variable of the HAPI-PCA with extremely high similarity and accuracy (Figure 11). However, the stage of the investigation, at this point, was not restricted to the Classical Least Squares method but expanded to encompass the Huber Regression method to adjust for the possible impacts of outliers and dispersal biases. As expected, the fundamental reason for implementing this method is to inspect the robustness of the coefficient matrix and observe how the matrix is protected against possible outliers within the dataset.

In contrast to traditional linear regression, Huber regression has a more robust form for dealing with outliers by introducing a loss function that is quadratic for small values and uses the absolute value function for large values. Hence, Huber regression not only has good methodological rigor for model development, but also has adequate methodology for testing the stability of results for the analysis of composite indices for socioeconomic variables, such as HAPI-PCA.

**Figure 13:**
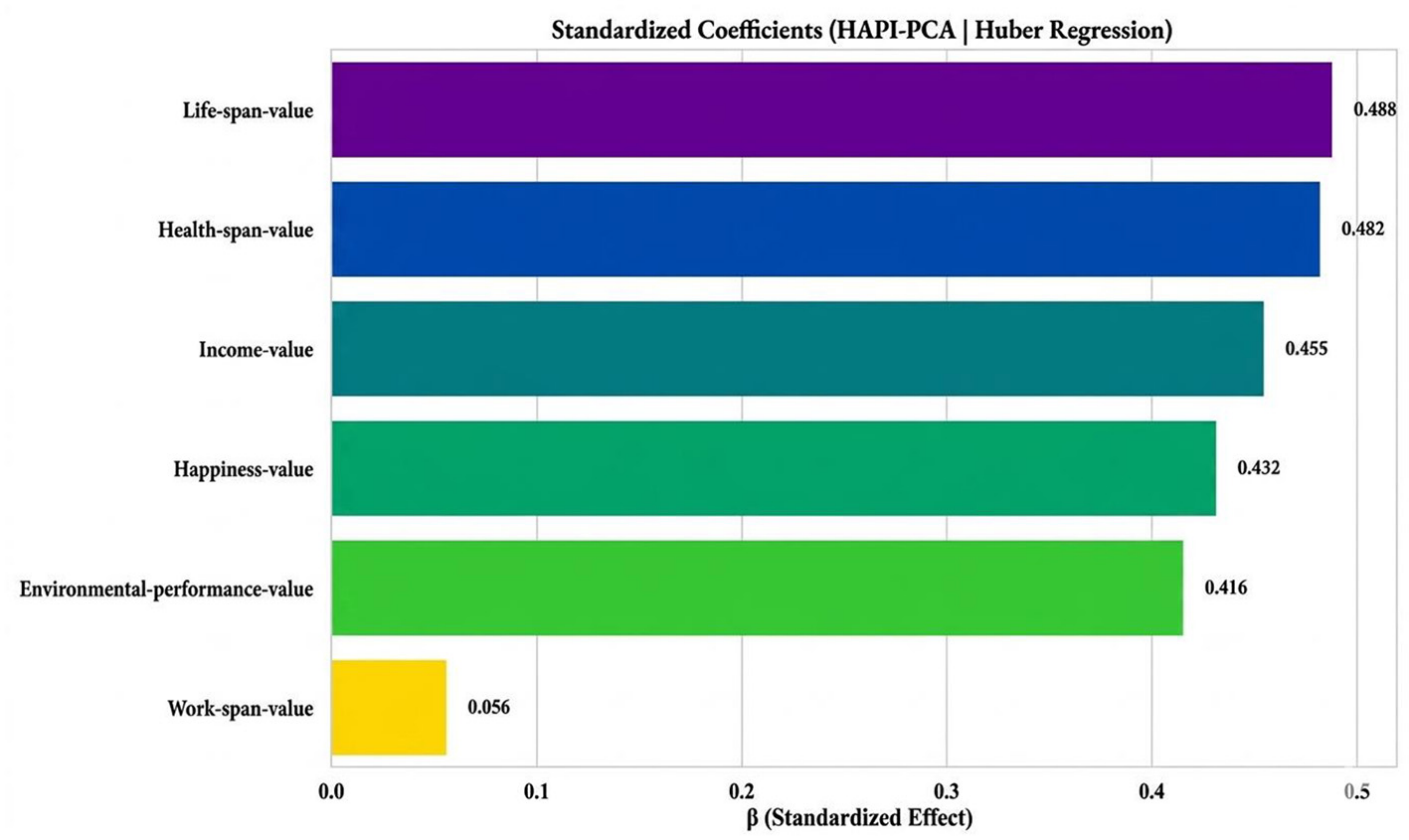
Standardized coefficients of the Huber regression model.

The coefficients from the Huber regression model when all variables are standardized are shown in Figure 13. Standardized beta coefficients (β values) enable direct comparison among the independent variables regarding their influence on HAPI-PCA.

The data depict that the Life-span Value (β = 0.4884) and Health-span Value (β = 0.4825) variables have the largest impact on HAPI-PCA. This depicts that the lifespan and health aspects have a pivotal role amongst the most influential factors of happiness. The Income Value (β = 0.4551) and Happiness Value (β = 0.4319) variables also proved a high impact value, which illustrates the pivotal role of economic conditions and happiness levels of individuals on the happiness index. The Environmental Performance Value (β = 0.4157) variable illustrated the supportive role of eco-sustainability on happiness levels, and the impact of Work-span Value (β = 0.0562) variables proved moderate.

**Figure 14:**
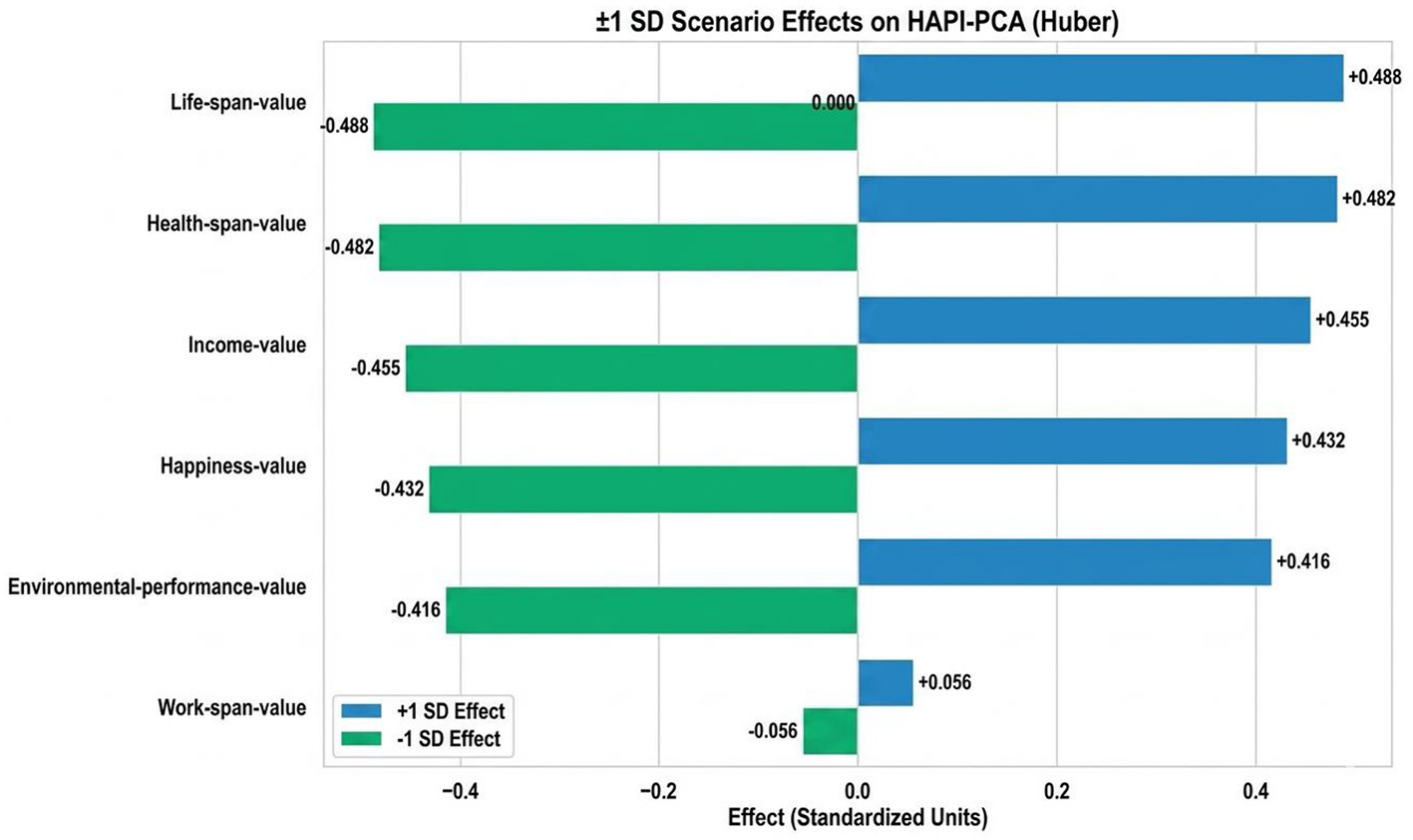
±1 standard deviation scenario effects on HAPI-PCA (Huber regression).

The scenario analysis for the ±1 standard deviation scenario with respect to the coefficients shown is presented below in Figure 14. The scenario analysis gives a better insight into how the marginal change in the variables affects HAPI-PCA positively or negatively.

The findings are fully consistent with the coefficient analyses; specifically, they confirm that variations in the indicators like life expectancy, health, income, and happiness have symmetrical and strong impacts on HAPI-PCA.

The following stage of the analysis was conducted by performing SHAP analysis to complement the SHAP values for interpreting the coefficients of the models from data. SHAP values provide an insight into interpreting the analysis carried out by computing the average marginal contribution of a variable to the model based on the principles of game theory.

**Figure 15:**
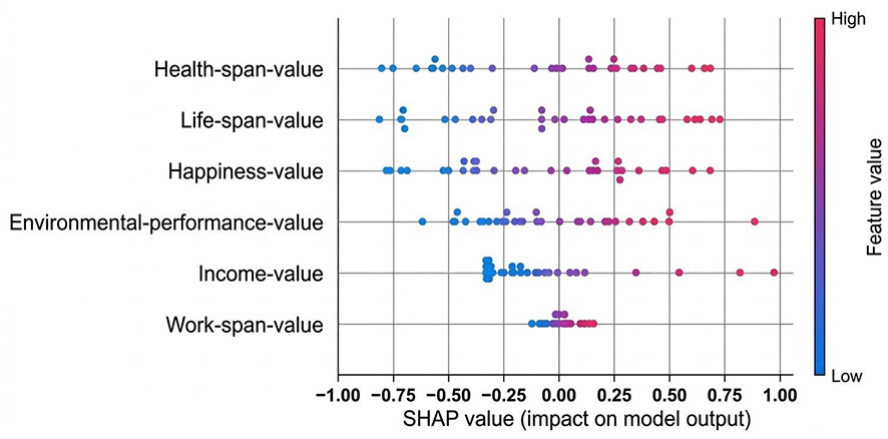
SHAP summary plot for the Huber regression model.

In the SHAP summary plot shown in Figure 15, global contributions towards the estimates by the HAPI-PCA are provided for the variables. The results are in line with the standardized coefficients obtained using Huber Regression.

**Figure 16:**
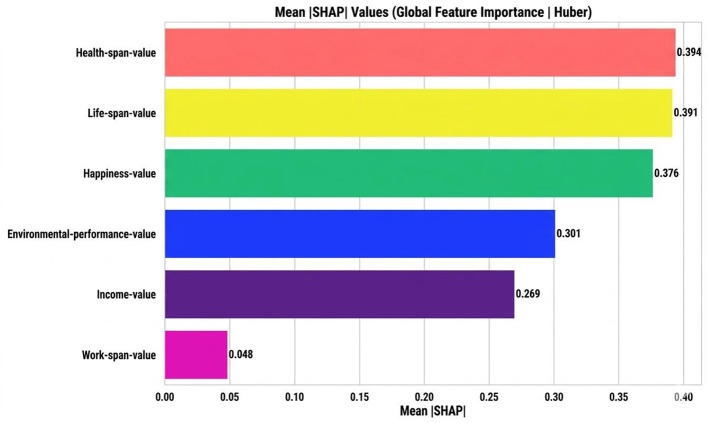
Mean absolute SHAP values (global feature importance).

The global variable significances calculated using the average absolute SHAP values are shown in Figure 16. “Life-span Value” and “Health-span Value” possess the greatest SHAP significance, thus proving that these features are the major deciding factors, not merely based on the coefficients, but on the complete data as well.

In general, this analysis done with the Huber Regression model proves to be rigorous in confirming the robustness and generality of the results acquired in the previous section. Figures 11–14 collectively show that linear models are not merely adept at making accurate predictions in the evaluation of composite indices like HAPI-PCA, but also are capable of making consistent and interpretable outcomes, and thus are better placed compared to nonlinear models in providing a methodological framework that aligns with the principles of science in the evaluation of indices.

In addition, it should be noted that the interpretation results obtained using linear analysis and SHAP values are not based on the weighting system generated through PCA alone. In order to further test the robustness of the results, an Equal Weight HAPI index (HAPI-EW) was created where equal weights were assigned to each dimension (*w* = 1/6). The ranking and dominance results obtained from this method are highly similar to those from the HAPI-PCA index.

### 4.4 Country-level structural decomposition and dimension-specific contributions to the HAPI index

From previous analysis, it has been shown that the composite index of the HAPI-PCA has a linear and index-based structure. The results of country-level structural decomposition analysis make it clear that the linear structure of the index is itself not a hypothetical construct but is firmly grounded in empirical reality. The country-specific contributions to the index computed on the basis of the standardized components disclose the structural composition of the total scores of the HAPI and make it evident that countries differing little in their overall welfare can actually enjoy different welfare production systems.

**Figure 17:**
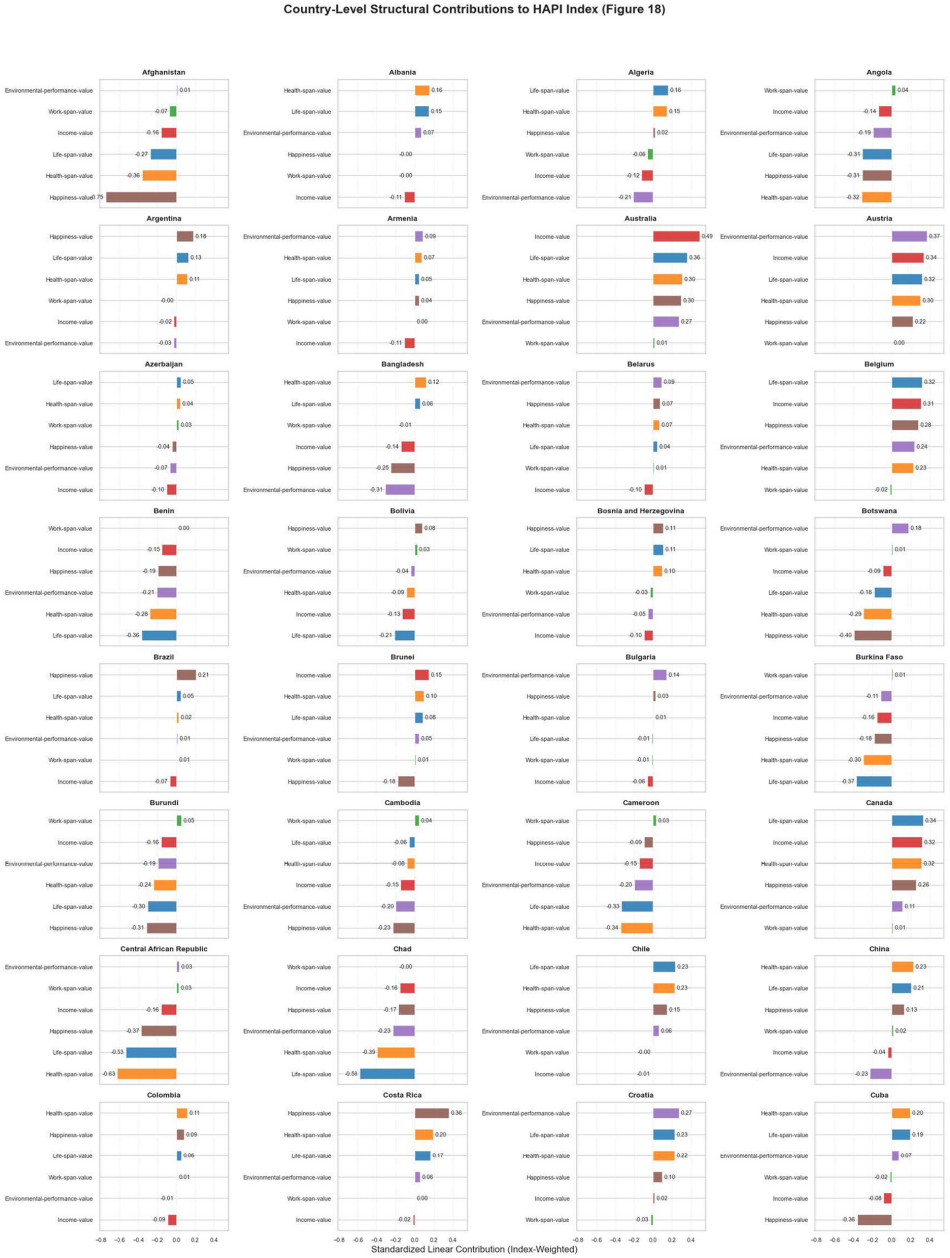
Country-level structural contributions to HAPI index (1–32).

**Figure 18:**
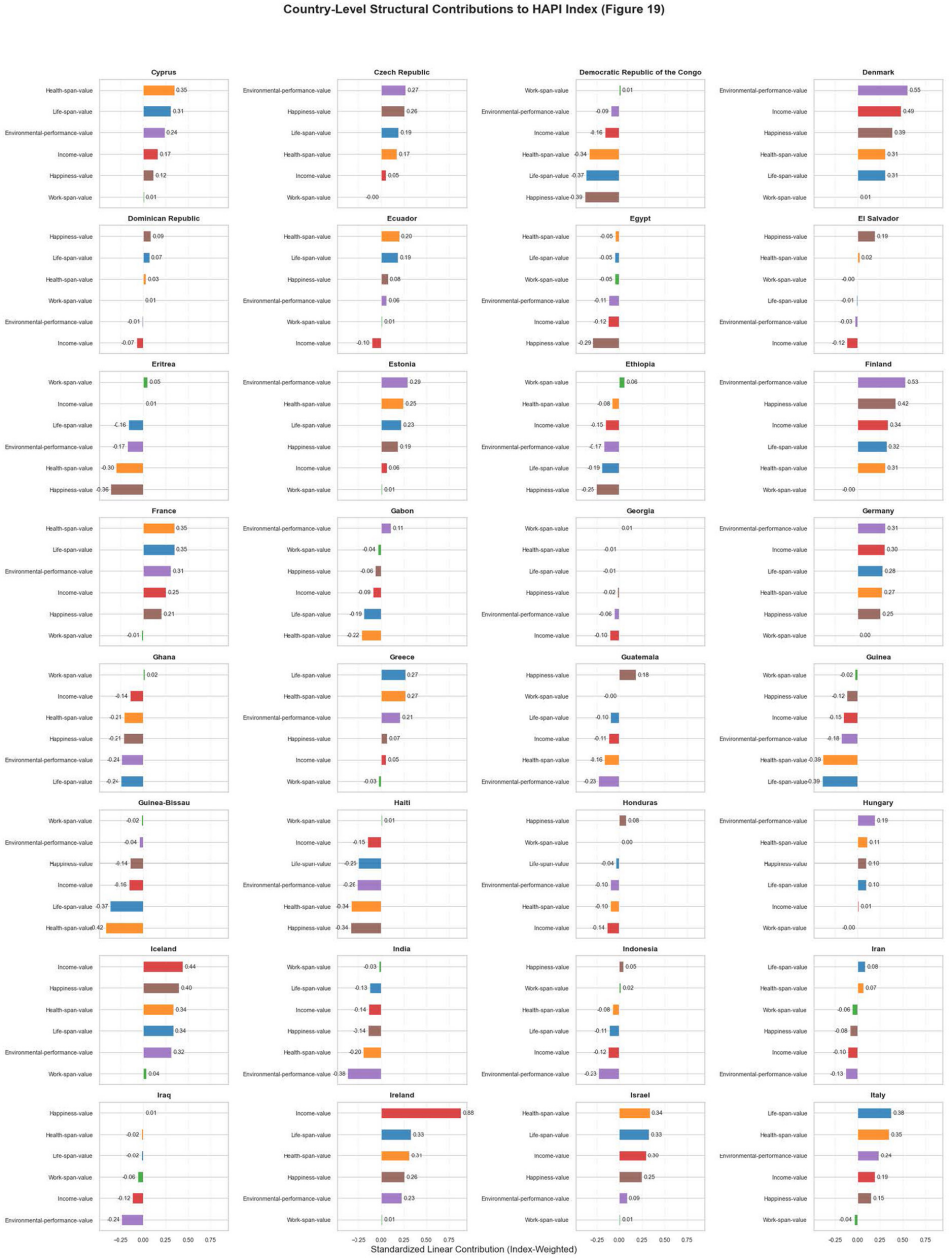
Country-level structural contributions to HAPI index (33–64).

In Figures 17, 18, the balance in the contribution structure, in both magnitude and direction, for the countries with strong HAPI performance can be noted. In the case of Finland, a strong and balanced positive contribution is achieved through the life expectancy (0.324) and health (0.309) indicators, and an extremely high value of 0.531 through environmental sustainability. Similarly, in the case of Denmark, a strong, balanced, positive contribution of 0.486 is generated through income, but an extremely high value of 0.553 is generated through environmental sustainability, ensuring that well-being is generated not only because of economic ability but also through a structure centered on sustainability. In countries like Switzerland and Luxembourg, a strong, balanced, positive contribution of 0.805 and an extremely high value of 1.016 is generated through income, but a strong, balanced, positive contribution through life expectancy (0.382 and 0.330) and health (0.363 and 0.330) indicators to counter the economic dominance. This clearly depicts in numbers how each of the well-being dimensions in these countries with strong HAPI performs in a balanced and complementary manner.

**Figure 19:**
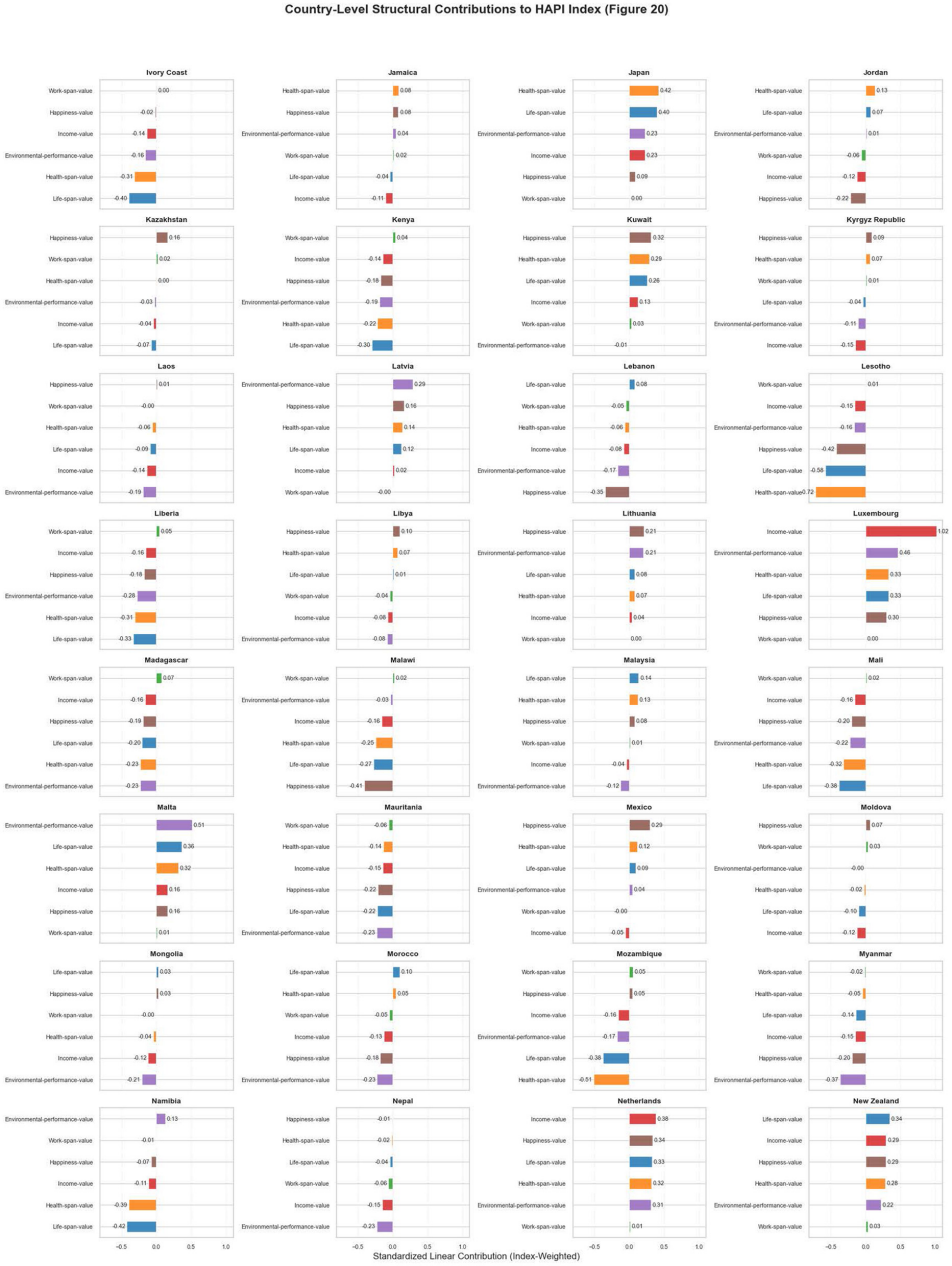
Country-level structural contributions to HAPI index (65–96).

**Figure 20:**
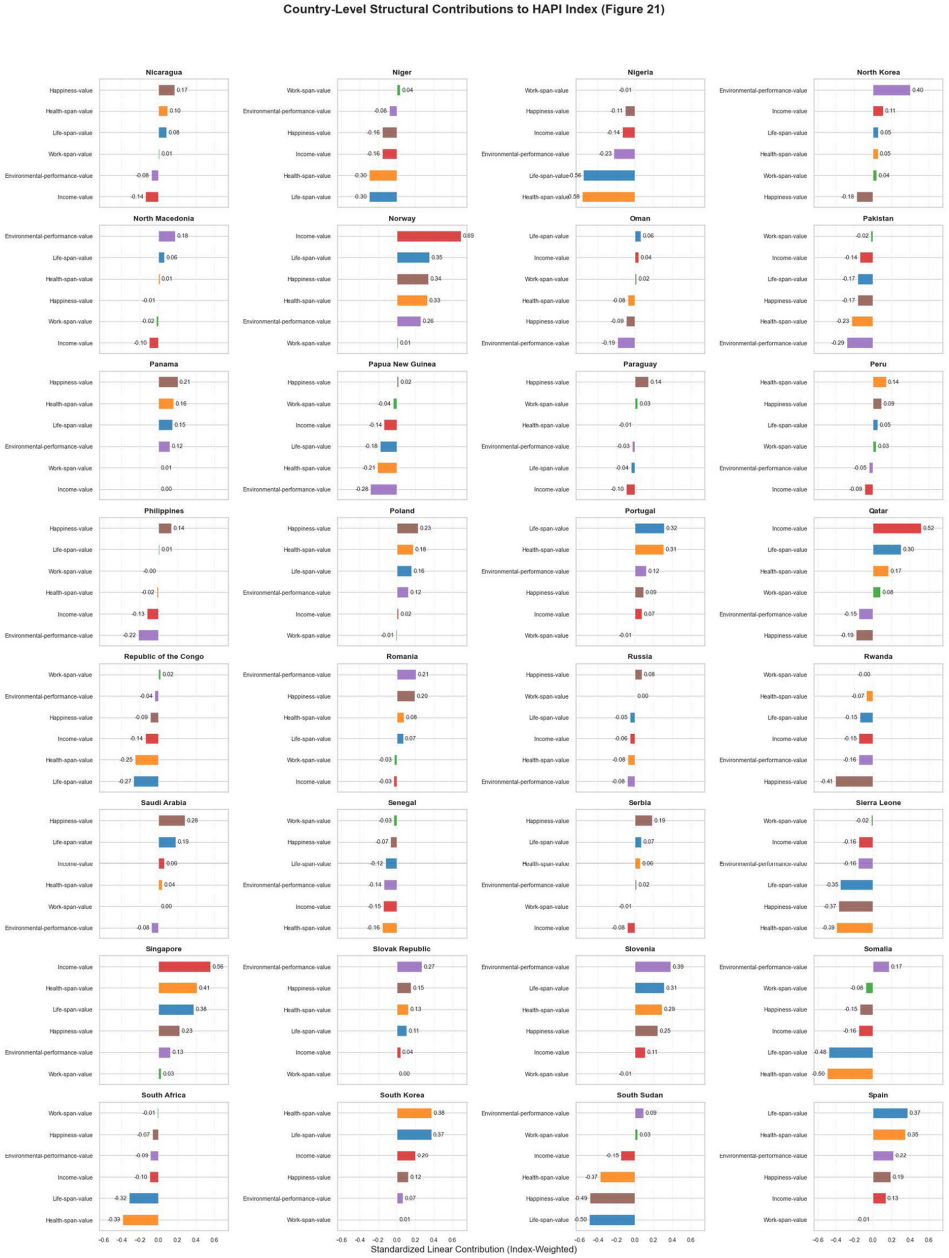
Country-level structural contributions to HAPI index (97–132).

While Figures 19, 20 demonstrate that the contribution structure of the countries with the HAPI performance scores around the moderate level are more fragmented and structurally unbalanced, in the case of Turkey, for example, while life expectancy equals 0.204 and health 0.217, which is significantly positive contributions, the strong negative value of environmental performance reaches −0.259, becoming the main limiting factor in the total HAPI score. The same type of structure is observed in China: even though life expectancy's share of 0.207 and health's of 0.228 are positive, environmental performance stays at −0.226 and income at −0.039, structurally indicating an unbalanced well-being. At the same time, in countries like Argentina and Brazil, happiness contributions reach 0.178 and 0.208, respectively, but the weak income and environmental performance indicators reveal that well-being is produced based on partial rather than holistic gains. Their total HAPI scores in these countries are the result of limited advantages in specific areas rather than the intrinsic consistency of well-being.

**Figure 21:**
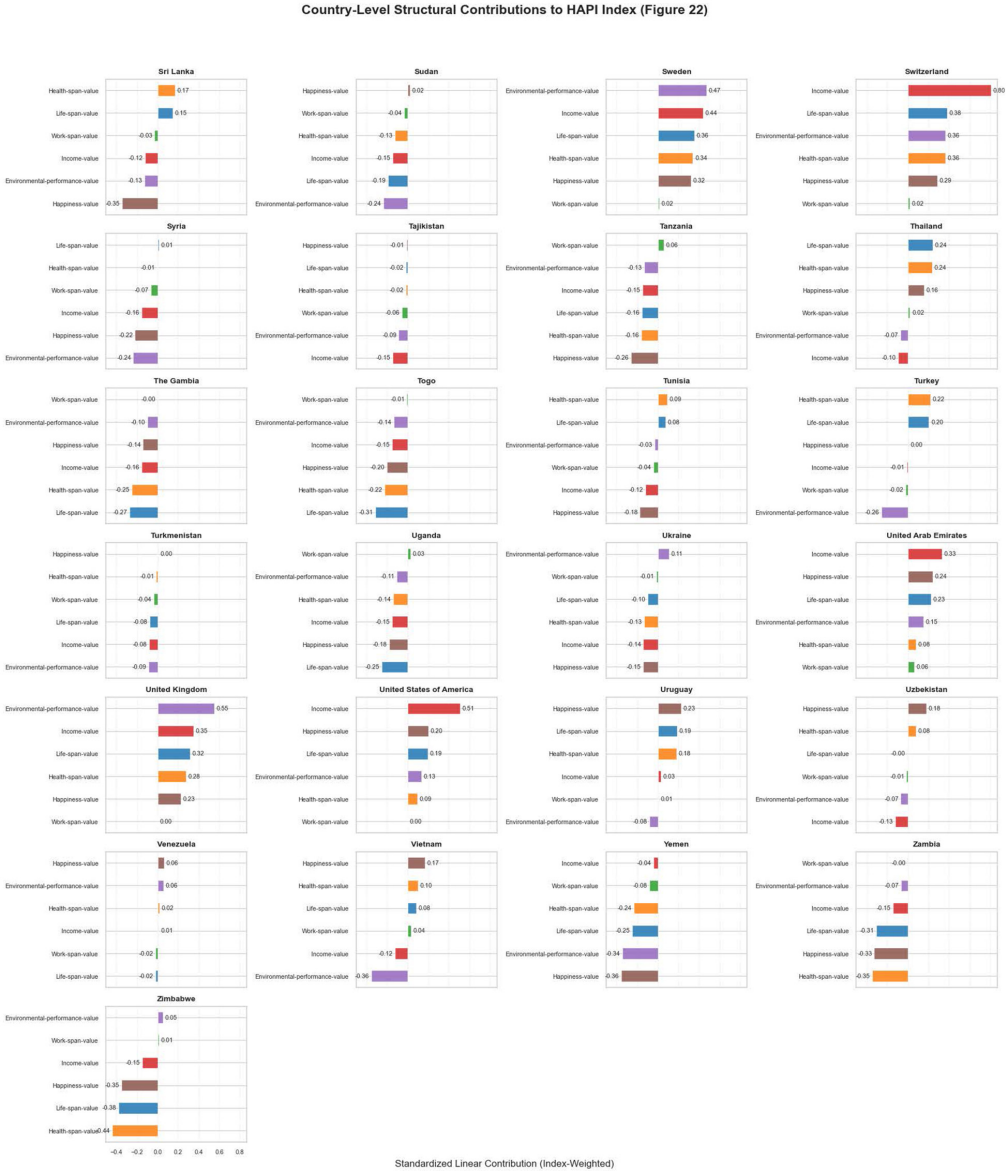
Country-level structural contributions to HAPI index (133–153).

It is evident from “Figure 21” that the contribution structures in regions with lower performances on the level of HAPI are dominated by negative values for almost all basic dimensions. In Afghanistan, the joint strong negative contribution of life expectancy (−0.275) by health (−0.360) and income (−0.159) and happiness (−0.749) indicators indicates that the absence of well-being is caused by multidimensional and deep-seated fundamental problems. In the Central African Republic, the very strong negative contribution of health (−0.627) and life expectancy (−0.533) indicates that the well-being architecture is, in the opinion of the HAPI indicator, structurally broken down. It is confirmed by the strong health contribution of −0.722 in the Lesotho country and the happiness contribution of −0.492 in the South Sudan country that the absence of well-being is characterized by the persistent fundamental structures and, besides the economic, also extends to the social and organizational levels. In Zambia, the health contribution of −0.349 and the happiness contribution of −0.329, and in Zimbabwe, the health contribution of −0.440 and the happiness contribution of −0.352, support this fundamental absence of well-being.

When taking a look at Figures 16–20 altogether, there is no doubt that the secondary role of the Work-span Indicator emerges concerning the cross-country differentiation process itself. The fact that the value close to zero for countries that categorize under different welfare systems like Germany (= 0.001), France (−0.015), Japan (= 0.004), Turkey (−0.024), and the USA (= 0.001) demonstrates that the key factors for the HAPI Index are defined by taking upon life expectancy measures, health conditions, environment factors, as well as subjective well-being measures, as opposed to working time measures.

More generally, the country-level structural decomposition shown in Figures 16–20 illustrates how the HAPI index is not a normative ranking tool but rather an explanatory analytical framework that clearly uncovers the structural elements through which countries achieve their observed welfare levels. No particular dimension of welfare is valued *a priori*; instead, different welfare regimes are rendered comparable by standardizing contributions. That even countries with similar total HAPI scores display very different contribution profiles clearly shows how individual score readings cannot be satisfactorily used for policy analysis and strongly confirms the need for considering welfare in conjunction with its structural components. Therefore, country-level structural decomposition should be regarded as an integral analytical step that reinforces conceptual consistency and empirical strength in the HAPI index.

This framework for decomposition also directly tackles one of the limitations of composite indices that use statistical weighting techniques like PCA and entropy weighting, specifically that of decreased interpretability at the dimension level. By breaking down the HAPI score for each country in terms of its contribution from each dimension, the current framework makes it possible for policymakers to determine which welfare dimensions are responsible for national performance.

### 4.5 Integrative assessment of global welfare: inequality, feature importance, and country clustering

**Figure 22:**
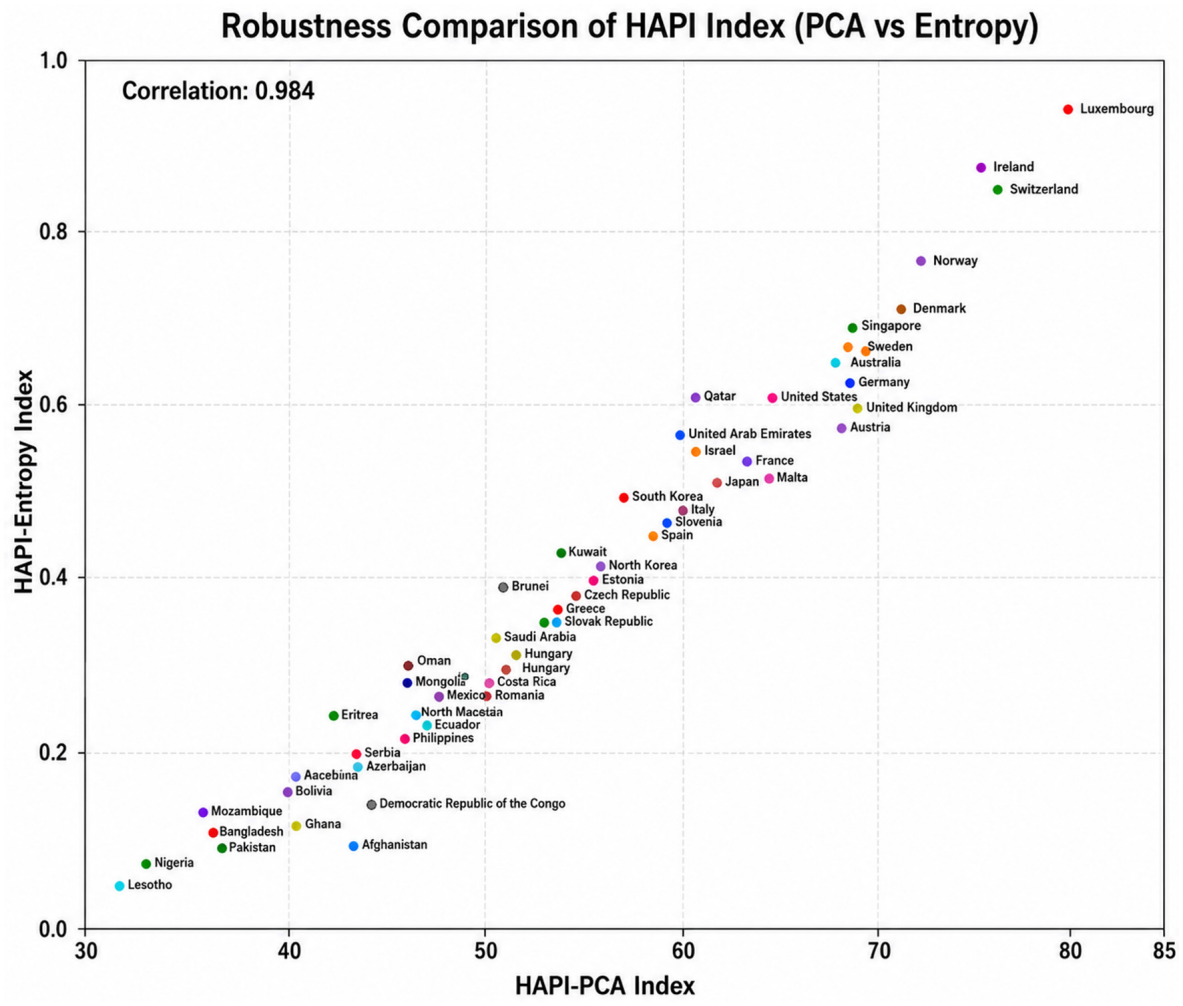
Robustness comparison of HAPI-PCA, HAPI-Entropy, and HAPI-EW (Equal Weight) indices across 153 countries, showing strong pairwise correlations and ranking stability under alternative weighting schemes.

After the structural analysis of the HAPI Index, the integrative analysis aimed to evaluate the inequalities, clustering, and feature importances of the HAPI Index across the countries. Figure 22 presents the scatter plot of the HAPI index with the use of the PCA and HAPI Entropy methods. This demonstrates the robustness of the HAPI Index, considering the linear and entropy-based approaches. The strong positive correlation (Pearson's *r* = 0.993, *p* < 0.001) between the two approaches indicates the high consistency of the results, implying that the linear and entropy-based approaches produce almost similar results in terms of the welfare levels of the countries. In order to further examine whether country rankings are sensitive to the weights used, another robustness check was conducted based on the Equal-Weight HAPI index (HAPI-EW), which assigns equal weights to all six dimensions of welfare, with each dimension having a weight of 1/6 (≈16.6%). Correlation analysis between HAPI-PCA and HAPI-EW shows that there is a very strong positive relationship between them (Pearson's *r* = 0.982, *p* < 0.001). It demonstrates that global welfare rankings are stable and insensitive to other weighting schemes. It can be seen that the findings are not affected by PCA optimization or entropy weighting scheme.

**Figure 23:**
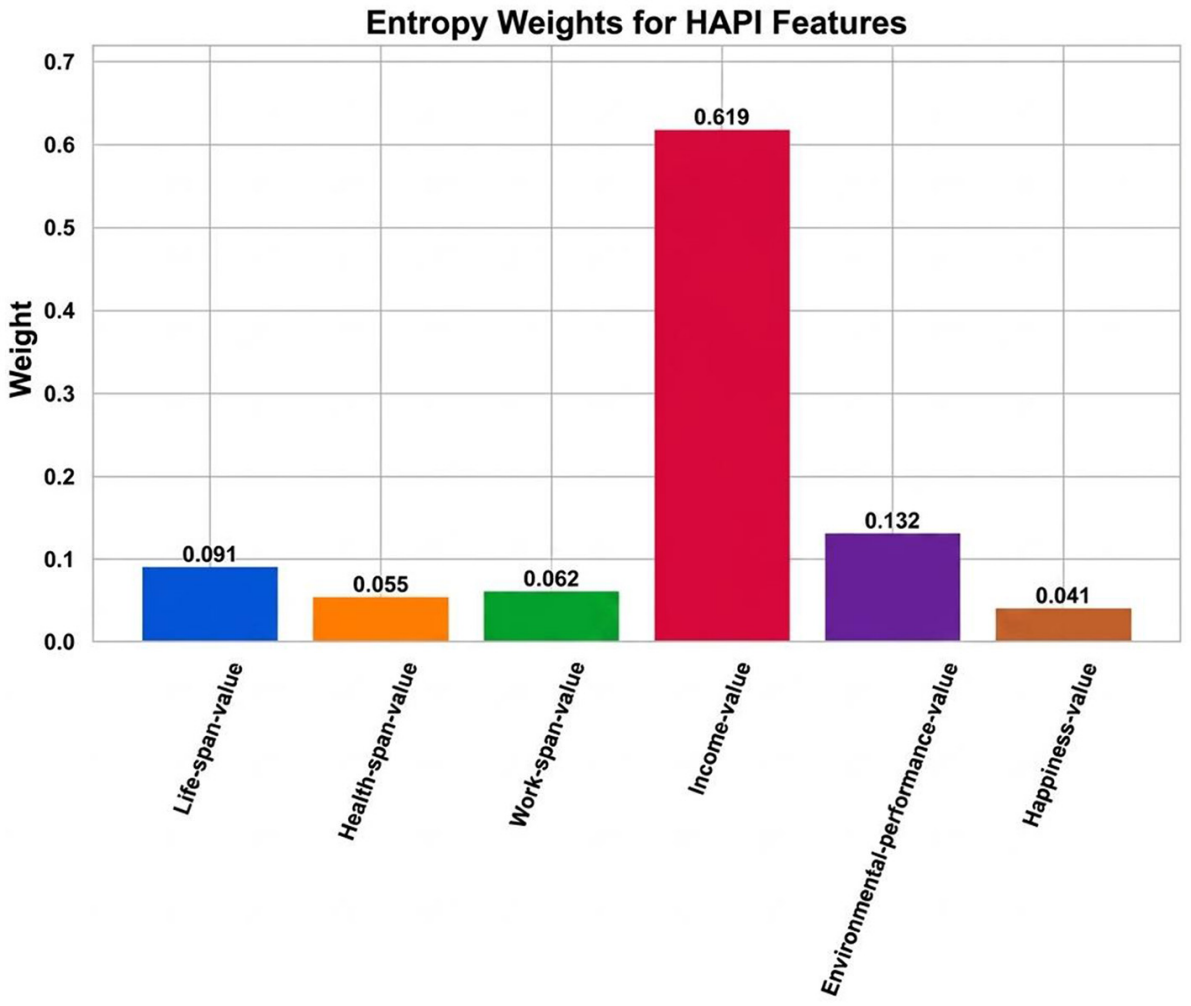
Entropy weights for HAPI Features, with emphasis on the relative importance of economic and environmental factors.

Figure 23 presents the entropy weights assigned to each feature during the HAPI Index calculation. The results indicate that the Income Value has the highest weight, approximately 0.619, followed by Environmental Performance with approximately 0.132, then Life-span Value with approximately 0.091, Work-span Value with approximately 0.062, Health-span Value with approximately 0.055, and finally Happiness Value with approximately 0.041.

**Figure 24:**
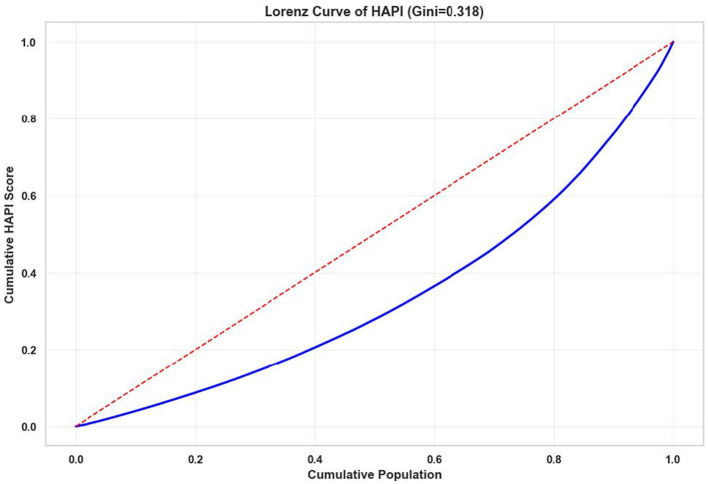
Lorenz curve for HAPI-Entropy scores with a Gini coefficient of 0.318.

The Lorenz curve in Figure 24 demonstrates the inequalities in the HAPI-Entropy scores. This curve indicates a moderate deviation from the line of perfect equality, which means the levels of welfare are not evenly distributed across the globe.

The Gini coefficient, which has been determined to be 0.318, indicates significant disparities in the levels of well-being among the countries. This means that even the countries with high HAPI scores may not be performing well in all dimensions.

**Figure 25:**
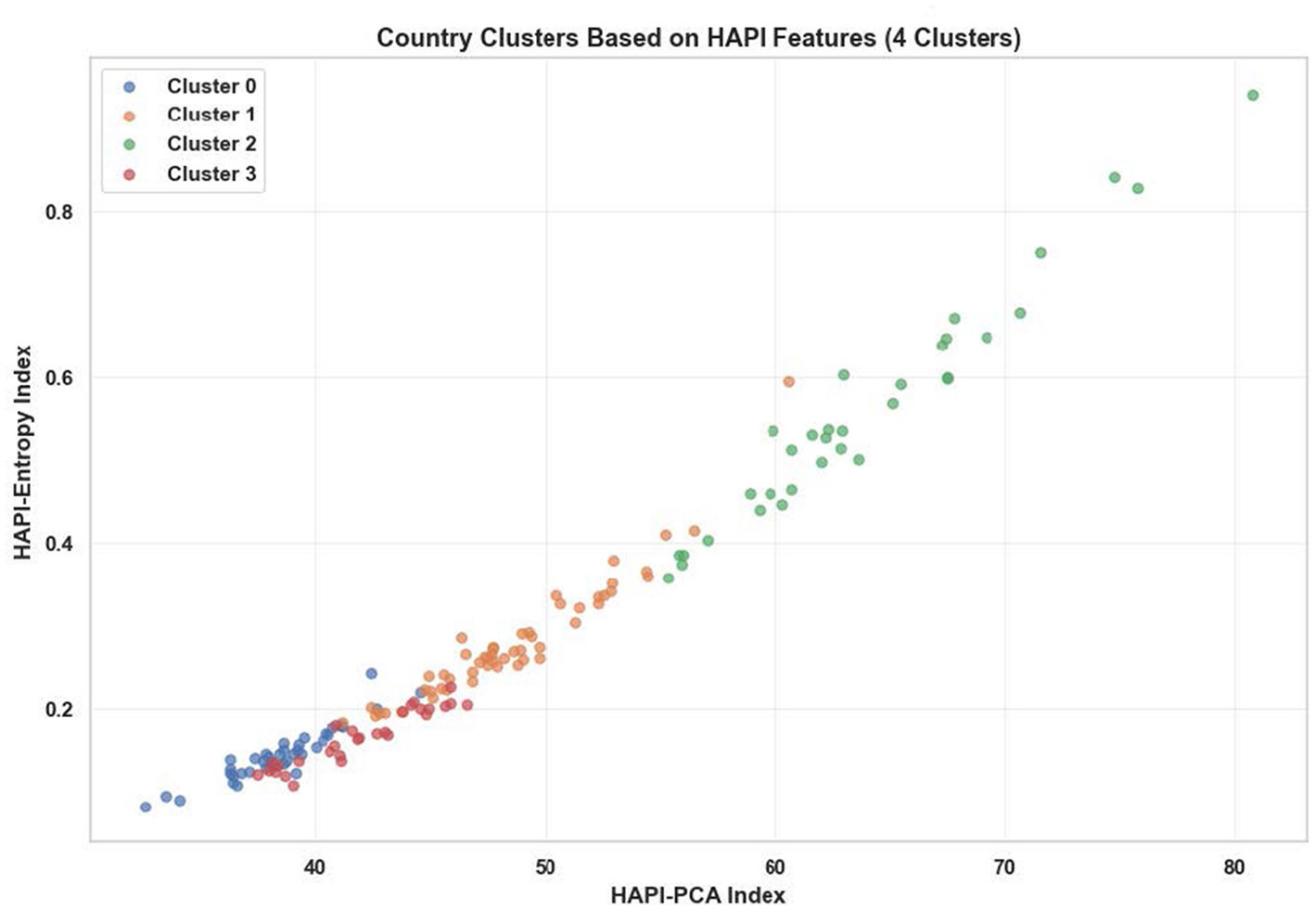
Country clusters based on HAPI features, emphasizing differences in structural characteristics of high-, medium-, and low-performing countries.

The clustering analysis with *K* = 4 creates a more detailed understanding of country-level welfare, as depicted in Figure 25. Countries with the lowest HAPI scores and minimal contribution in critical dimensions such as life expectancy, health, income, and environment are included in Cluster 0. Countries with average HAPI scores and mixed contributions, implying mixed performance in welfare dimensions, are included in Cluster 1. Countries with high performance and balanced contribution in most dimensions are included in Cluster 2, while those with low-to-medium HAPI scores and specific weaknesses in some dimensions are included in Cluster 3.

Generally, Figures 21–24 above show that the HAPI approach is indeed methodologically sound regardless of the kind of weighting used, such as PCA weighting, entropy weighting, and equal weighting. The highly correlated results in the different specifications used prove that the HAPI approach is indeed capable of detecting stable welfare structures and not artifacts of a particular weighting procedure. In addition, the use of scatter plots, entropy weights, Lorenz curves, clustering analysis, and structural decomposition enables one to analyze welfare inequalities globally.

## 5 Conclusion and policy implications

The present study proposes a multidimensional framework for the analysis of healthy aging. Unlike the conventional one-dimensional approach, the present study considers the economic and environmental well-being of the individual along with their subjective well-being. This is because the concept of healthy aging is not a mere outcome. Rather, it is a structural process through which the social, economic, and environmental factors collectively contribute to the experiences of individuals over their lifespan (3). In this context, the functional capacity-centered approach proposed by the World Health Organization seems to be in alignment with the present study. It emphasizes that the evaluation of healthy aging must include not only the lifespan of the individual but also their functional capacity and well-being (58).

Prevention index (HAPI), offer strong support for the rationale underlying the importance of multidimensional measurement in healthy aging research. Past research has established that functional ability, intrinsic capacity, and healthy aging indices are major determinants of mortality and dependency risk (2). Similarly, the PCA used in this research shows that healthy aging from a macro perspective is not random and has a structured pattern.

Moreover, regression analyses show that, apart from the well-known linear effects, healthy aging is highly stable and predictable on a country level. Classical regression analysis, Huber regression analysis, and Bayesian regression analysis all demonstrate that structural and institutional factors are dominant in healthy aging, as opposed to individual preferences and lifestyles. These results are in line with the overall literature on lifelong effects of socioeconomic conditions and the inadequacy of health-focused interventions.

The results of the cluster analysis of the data through the K-Means algorithm support the findings of the heterogeneous nature of the healthy aging profile among the countries. Similar to the findings of the multi-component indices of healthy aging, such as the ATHLOS Healthy Aging Scale (11), the results of the cluster analysis show that the countries are grouped into distinct clusters based on their economic, environmental, and well-being factors. The significant negative correlations and distances among the clusters show that the factors affecting healthy aging cannot be attributed only to economic development; therefore, it is necessary to consider additional factors affecting healthy aging, including environmental quality and subjective well-being. These findings support the need for a different policy approach in each of the clusters since the structural context of healthy aging in each country may differ (59, 60).

The results of the SHAP-based explainability analysis provide further validation of the multi-dimensional theoretical framework. Variables related to lifespan and healthy lifespan have a high contribution to healthy aging outcomes as expected. However, the high and consistent contribution of income and happiness variables indicates that healthy aging outcomes go far beyond the performance of healthcare systems. Similarly, the consistent contribution of environmental performance indicates the indirect yet significant role of sustainable environmental conditions in healthy aging outcomes (4, 58).

It is important to note that the results from the simulations should be taken to mean that the changes are associational and not causal since any improvement shown depends on model-generated sensitivity analysis of the HAPI framework under some theoretical changes.

Any improvements in any of the dimensions of the HAPI will have substantial effects on healthy aging results from the simulation study results. Nevertheless, what has been achieved from this simulation exercise is associational and cannot be regarded as causal in nature. It means that a healthcare policy that focuses only on healthcare expenditures is insufficient. There is need for a comprehensive life-long policy that is intersectoral covering economic security, environmental factors, and subjective well-being, as argued by Strandberg et al. (16).

Also, the findings suggest the use of cluster analysis and artificial intelligence in policy-making for better outcomes both nationally and regionally.

Integrative analysis of HAPI Index using PCA and Entropy approach strengthens its reliability and brings out the welfare inequalities in the world. Economic indicators and environmental performance have the greatest influence on the HAPI Index, while lifespan, health, workspan, and happiness have a relatively moderate effect on it. It confirms that fact that healthy aging is influenced by economic, environmental, and social factors. Inequalities in the HAPI Index are confirmed since Gini coefficient of HAPI Index comes out to be 0.318. The cluster analysis results confirm the fact of welfare inequalities in the HAPI index. This shows that there is no guarantee of obtaining high scores in all dimensions if a country scores high in the HAPI Index.

In conclusion, it is worth noting that the study makes a significant contribution to methodological and theoretical knowledge of healthy aging by combining PCA, entropy-weighted HAPI, regression, clustering, and SHAP explainability techniques in a single framework. The results clearly show that healthy aging is a social and structural construct rather than an individual construct. The addition of entropy weighting techniques highlights the importance of economic security, environmental quality, and subjective well-being, in addition to the traditional concepts of lifespan and health-span. The clustering results show that there are interesting patterns of heterogeneity across the nations, and it is worth noting that for successful policy interventions, a multidimensional, evidence-based, and country-specific approach is necessary, as a one-size-fits-all policy is unlikely to be effective.

## 6 Limitations and future research directions

The current study is faced with certain limitations that have to be mentioned. First, aggregated information at the country level means that the impact of micro-level socio-demographic factors on healthy aging cannot be assessed. Secondly, the multi-dimensional approach applied when constructing the HAPI index does not account for contextual factors, including culture, social capital, the quality of the healthcare system, and regional access to services. Thirdly, being a cross-sectional study, the current research does not show any cause-and-effect relationship; hence the findings of regression analysis, cluster analysis, and simulations are mere correlations and should not be interpreted causally. Fourthly, the findings of machine learning and SHAP analysis depend greatly on the chosen algorithm and model specification.

## Data Availability

The datasets presented in this study can be found in online repositories. The names of the repository/repositories and accession number(s) can be found below: https://github.com/Sadullah4535/Healthy-Aging-and-Preventive-/upload/main.
